# Investigation of the transcriptomic response in Atlantic salmon (*Salmo salar*) gill exposed to *Paramoeba perurans* during early onset of disease

**DOI:** 10.1038/s41598-021-99996-1

**Published:** 2021-10-19

**Authors:** Anita Talbot, Laura Gargan, Grainne Moran, Louis Prudent, Ian O’Connor, Luca Mirimin, Jens Carlsson, Eugene MacCarthy

**Affiliations:** 1 Galway Mayo Institute of Technology, Galway, Ireland; 2 University College Dublin, Dublin 4, Ireland

**Keywords:** Molecular biology, Transcriptomics, Infection

## Abstract

Amoebic Gill Disease (AGD), caused by the protozoan extracellular parasite *Paramoeba perurans* (*P. perurans*) is a disease affecting Atlantic salmon (*Salmo salar*). This study investigated the gill transcriptomic profile of pre-clinical AGD using RNA-sequencing (RNA-seq) technology. RNA-seq libraries generated at 0, 4, 7, 14 and 16 days post infection (dpi) identified 19,251 differentially expressed genes (DEGs) of which 56.2% were up-regulated. DEGs mapped to 224 Gene Ontology (GO) terms including 140 biological processes (BP), 45 cellular components (CC), and 39 molecular functions (MF). A total of 27 reference pathways in the Kyoto Encyclopedia of Genes and Genomes (KEGG) and 15 Reactome gene sets were identified. The RNA-seq data was validated using real-time, quantitative PCR (qPCR). A host immune response though the activation of complement and the acute phase genes was evident at 7 dpi, with a concurrent immune suppression involving cytokine signalling, notably in interleukins, interferon regulatory factors and tumour necrosis factor-alpha (*tnf-*α) genes. Down-regulated gene expression with involvement in receptor signalling pathways (NOD-like, Toll-like and RIG-1) were also identified. The results of this study support the theory that *P. perurans* can evade immune surveillance during the initial stages of gill colonisation through interference of signal transduction pathways.

## Introduction

Amoebic Gill Disease (AGD) is a gill disease of marine cultured Atlantic salmon, caused by the free-living protozoan *Paramoeba perurans (P. perurans)*^1^ also known as *Neoparamoeba perurans*. Among the parasitic conditions affecting gill health, AGD is the most significant in terms of prevalence and economic impact^2^. First described in Tasmania in 1985^1^, AGD is now present in most Atlantic salmon producing countries^2^ and affects fish during the marine grow out cycle of production at sea temperatures ranging from 7 to 20 °C^2^. Macroscopically, AGD lesions are visible as white raised mucoid patches on the gill, due to increased mucus production by mucous cells. The colonisation of the gills by *P. perurans* initiates a tri-phasic host response which includes a localised reaction to parasite attachment, non-specific immuno-regulatory cell infiltration and advanced hyperplasia with epithelial stratification^3^. Proliferation and fusion of the lamellar epithelium decreases the functional gill surface area and increases the diffusion distance in the water-blood barrier for oxygen transfer. Consequently, AGD-affected individuals experience respiratory disturbances^4^. Microscopically, fresh gill smears can be used to detect the presence of amoebae. Additionally, histological examination of AGD gill tissue is used to confirm hyperplasia, lamellar fusion, the presence of lamellar vesicles in addition to the presence of amoeba, while species identification can be confirmed using a real-time PCR (qPCR) assay^5^. A widely used practical method for determining the presence and severity of AGD is the gross gill score^6^ (0 indicating no AGD, and 5 signifying extensive disease) across each side of all 8 gills. In the absence of a vaccine, current AGD treatments are limited to freshwater or hydrogen peroxide baths, which remove the amoebae from affected gills, and reduce gill mucus viscosity^3,7^. Bath treatments can cause stress and result in mortalities, and multiple treatments may be required during the grow-out production cycle, due to high re-infection rates. Gill scoring is the primary method employed to monitor AGD^6^ and is often used to initiate treatment. However, a recent study looking at gene expression, gross morphology and histopathology as tools for monitoring proliferate gill disease (PGD) in salmon reported a lack of association between gill score and gene expression or histology^8^. Previous AGD gill gene expression studies have utilised numerous molecular techniques including microarray analysis^9–11^, polymerase chain reaction (PCR)^12–17^, and more recently RNA-seq^18,19^ to investigate host response expression profiles. Although results can vary depending on the dose and virulence of the *P. perurans*, fish size, duration of infection and use of lesion or non-lesion specific gill tissue, these earlier studies found a localised immune suppression with genes involved in Th1, Th17 and Tregs pathways being down-regulated^12^, while pro-inflammatory cytokines (Il-1β)^13^,and Th2 cytokines (il4/13a and il4/13b2)^12,14^ were up-regulated. Mucus production is increased with AGD and investigation into mucin genes expression found mucin 5 to be up-regulated ^14^. The differential expression of the superoxide dismutase and catalase genes involved in oxidative stress has also been reported in late stage AGD^20^. The down-regulation of tumour suppressor p53 (p53) has been proposed as one of the mechanisms underlying cell proliferation in AGD^21^. Resistance to AGD has a moderate heritable genetic component and a recent study combining genome wide association analysis with RNA-seq gene expression profiling in the gill, reported the down-regulation of immune system components during the initial stages of the infection, while genes involved in cell adhesion processes were found to be up-regulated^18^. The involvement of the interferon response, systemic inflammation and apoptosis have also been associated with AGD resistance^22^. More recently, a dual RNA-seq study investigating the host-parasite interaction identified two transcription factors, znfOZF-like and znf70-like, with involvement in the immune response, cellular proliferation and invasion to be differentially expressed in response to AGD. The aim of this study was to utilise RNA-seq technology to investigate further the gill transcriptome, subsequent to *P. perurans* colonisation but in advance of mucoid lesion development, in AGD-affected fish, in order to identify biomarkers of early-stage disease.

## Results

### Gill score

Clinical symptoms of AGD were determined by macroscopic examination of the intact gills in euthanised fish and were scored according to Taylor et al.^6^. Prior to sampling, 3 fish were taken from each duplicate control and AGD-affected tanks, and all 6 fish placed in an anaesthetic bath. Naïve control fish (6) and experimental fish (6) were sampled at each time point (0, 4, 7, 14 and 16 dpi) and all fish were gill scored. No lesions were evident on the gills in AGD-affected groups at 4 and 7 dpi, with 3/6 fish at gill score 1 at 14 dpi. At 16 dpi, all six fish had a gill score of between 1 and 2.

### Detection of *P. perurans* DNA

Gill tissue from six naïve control, and six AGD-affected fish were analysed by qPCR for the presence of *P. perurans* DNA at 0, 4, 7, 14 and 16 dpi. The higher the crossing threshold value (Ct), the less *P. perurans* DNA that was detected. The qPCR was negative for the naïve control fish at all five sampling time points. The Ct values in the AGD-affected fish at 4 dpi were 34.6 ± 0.47, n = 5 (1 fish had a negative result with no *P. perurans* DNA detected), at 7 dpi was Ct 31.8 ± 2.7, n = 6, at 14 dpi the Ct was 28.8 + 1.5, and at 16 dpi the Ct was 29.4 + 1.7. The qPCR results indicate an increasing amoebic load, based on the level of *P. perurans* DNA detected, on the gill from 4 to 16 dpi.

### Histopathology analysis

**Figure 1:**
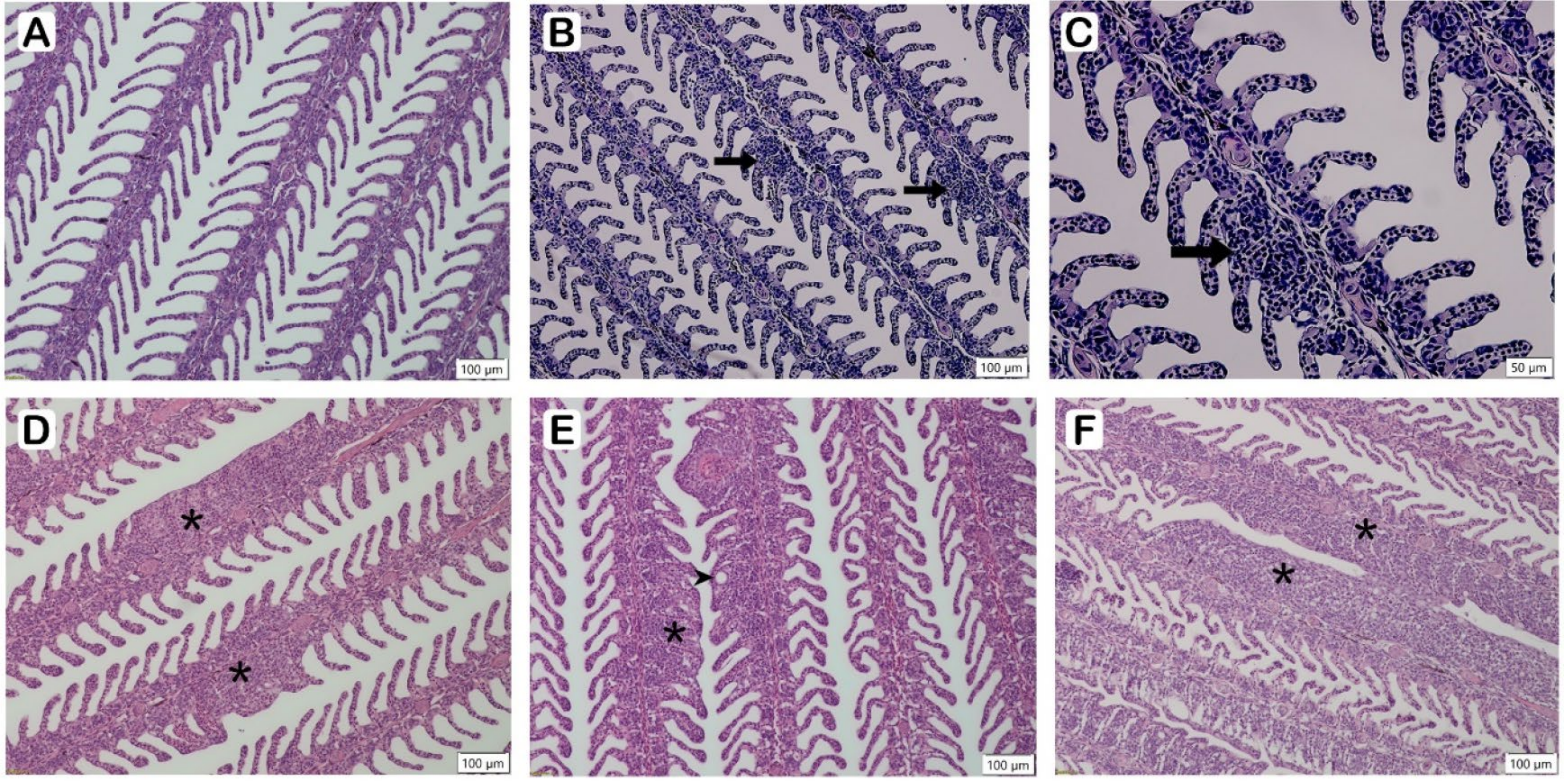
Histology in AGD-affected gills at 0 dpi, (**A**) 4 dpi, (**B**,**C**) 7 dpi, (**D**) 14 dpi, (**E**) and 16 dpi (**F**) in AGD-affected gill tissue. Normal gill naïve control fish showing individual gill filaments with lamellae (**A**). Some small areas of mild epithelial hyperplasia is evident at 4 dpi, indicated by arrows, (**B**), also shown at a higher magnification (**C**). More extensive hyperplasia and lamellar fusion, indicative of AGD, can be seen at 7dpi (**D**) and is indicated by asterisks. The increasing hyperplasia, lamellar fusion (asterisks) with the formation of lamellar vesicles (arrowhead) is evident at 14 dpi (**E**). At 16 dpi (**F**) there is extensive lamellae fusion in adjoining gill filaments (asterisks). Slides were stained with H&E and the scale is indicated on each image.

Gills were examined microscopically following staining with haematoxylin/eosin (Fig. 1). Some small areas of mild epithelial hyperplasia was evident at 4 dpi, while more extensive hyperplasia and lamellar fusion can be seen at 7dpi. Substantial hyperplasia and lamellar fusion with the formation of lamellar vesicles, indicative of AGD, were evident at 14 dpi and 16 dpi.

### Mapping RNA-seq reads to reference genome

Information related to the number of reads and alignments to the *Salmo salar* reference genome ICSASG_v2 (GenBank: GCF_000233375.1)^23^ for the 6 fish from each of the 5 sampling time points (0, 4, 7, 14 and 16 dpi) is presented in Supplementary Table S1 online. The sequencing depth ranged from 21.7 to 35.4 M raw reads. The number of clean, paired reads ranged from 19.8 to 32.9 M (88.4% to 94.7%). Assigned fragments in HISAT2 ranged from 18.7 to 31.8 M (91% to 97%) and the number of assigned feature counts ranged from 14.7 to 25.2 M.

### Sample similarity

Principal component analysis (PCA) was generated to visualise the relationships between all samples, as well as samples by time point (Supplementary Fig. S1 online). The similarity between samples from thirty-six RNA-seq libraries (6 fish × 6 groups, including 2 groups from T0) was analysed. Only one of the control libraries (T0_GCRL) was used in the pairwise comparisons. The variation in the PC1 was 70%, and in PC2 was 4%. From the PCA plot, it was evident that four of the six AGD-affected samples at 7 dpi clustered together, while one sample aligned with the earlier time points, and one sample aligning with the later time points.

### Differentially expressed genes (DEGs)

**Figure 2:**
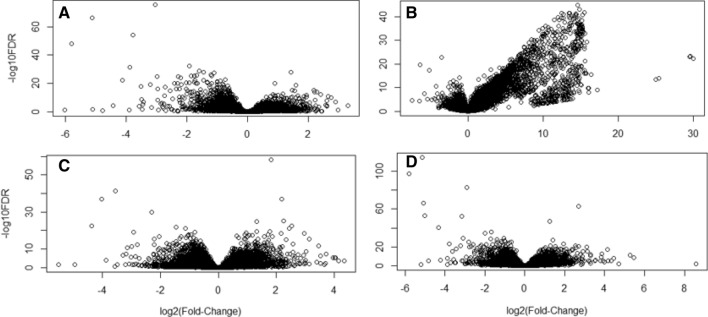
Volcano plots illustrating the pattern of differential gene expression over time. Plots represent 4 dpi (**A**), 7 dpi (**B**), 14dpi (**C**) and 16 dpi (**D**). The X-axis represents the log_2_FC in expression with negative numbers representing down-regulated expression and positive numbers representing up-regulated expression. The Y-axis is the *p*-value adjusted for or the false discovery rate (FDR < 0.05) to the negative log base 10. The pattern of expression was different at 7 dpi with more genes being differentially expressed, more genes being upregulated and with a higher log_2_FC compared to the other time-points.

Differentially expressed genes were identified at each of the four experimental time points (4, 7, 14 and 16 dpi) relative to naïve control fish (0 dpi). The number of genes differentially regulated in the AGD-affected gill tissue was 19,251 of which 56.2% were up-regulated over the course of the AGD trial. The pattern of gene expression at 4, 14 and 16 dpi identified more genes down-regulated (61.6%, 52.4%, and 54.7% respectively) with the majority of genes having a log_2_fold change (log_2_FC) expression between − 2 and + 2. The pattern of gene expression shifted more toward gene up-regulation at 7 dpi (59.6% vs 40.4%) and a greater increase in the log_2_fold expression with most genes falling between a log_2_FC of − 2 and + 15 (Fig. 2).

**Figure 3:**
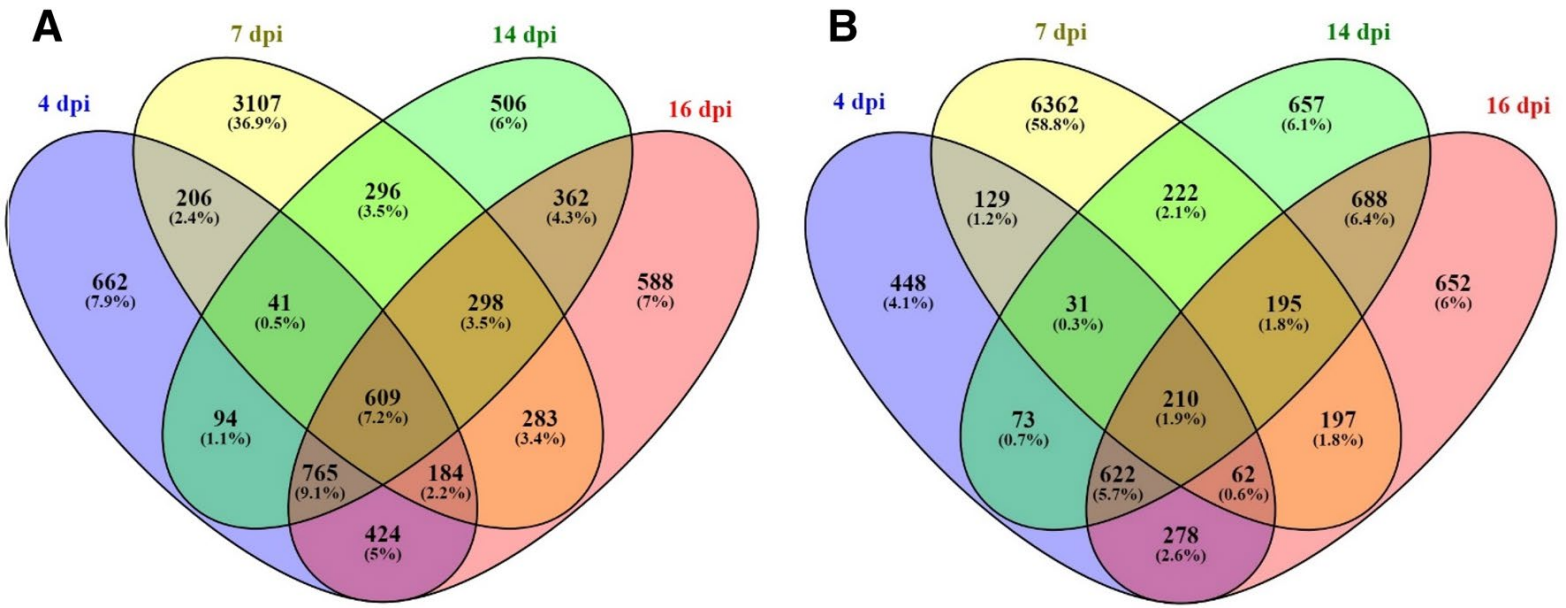
The distribution of differentially expresses genes in AGD-affected gill. The four-way Venn diagrams show the number and percentage of genes (**A**) down-regulated and (**B**) up-regulated at 4, 7, 14 and 16 dpi. Genes with a *p*-value adjusted for a false discovery rate (FDR) < 0.05 were assigned as differentially expressed in DESeq.

Of the total number of differentially regulated genes (*Salmo salar* gene IDs) identified by RNA-seq, 8425 were down-regulated and 10,826 up-regulated. A number of genes were differentially expressed at only one time point; 4 dpi; 1110 genes with 662 down- and 448 up-regulated, 7 dpi; 9469 genes with 3107 down- and 6362 up-regulated, 14 dpi; 1163 genes with 506 down- and 657 up-regulated, 16 dpi; 1240 genes with 588 down- and 652 up-regulated. There were 609 down-regulated genes and 210 up-regulated common to all four time points (Fig. 3).

### Validation of RNA-seq data using qPCR

Ten genes from the gill RNA-seq data were selected for qPCR validation across all sampling time points (Table 1). All analysis was carried out on individual fish gill samples (n = 30). The qPCR pairwise comparisons matched those carried out in the RNA-seq study. Genes included *acod1: aconitate decarboxylase 1* (also known as *irg1*), *cath1: cathelicidin 1*, *clra*: *c type lectin receptor a*, *cc4: c–c motif chemokine 4-like*, *lect2: leukocyte cell-derived chemotaxin 2-like*, *il-8: interleukin-8-like*, *il-17a/f1: interleukin-17a/f1*, *nos2: nitric oxide synthase 2, inducible*, *ptx: pentraxin-related protein-like*, *sap: serum amyloid P-component-like*. These genes were selected from the RNA-seq data for validation based on their expression at more than one time point and their involvement in the immune response (Table 1). The results of the qPCR confirmed that *acod1*, *cc4*, *il-17a/f1* and *nos2*, and *ptx3* were down-regulated at every time point. *Cath1* was down-regulated at 4, 7 and 16 dpi. Two genes, *clra* and *lect2* showed the same pattern of expression being greatly up-regulated at 7 dpi, while being down-regulated at the other time points. *Il-8* was down-regulated at 7 dpi and up at 16 dpi. *Sap* was up-regulated at 4, 14 and again at 16 dpi (Table 1). To use the qPCR data to validate the RNA-seq data, a Spearman correlation analysis was performed using the qPCR log_2_FC average of 6 individual fish for each of the ten genes. The correlation coefficient rho (ρ) and statistical significance (*p*) was 4 dpi: ρ = 0.91, *p* < 0.0002, 7 dpi: ρ = 0.89, *p* < 0.0003, 14 dpi: ρ = 0.78, *p* < 0.008 and 16 dpi: ρ = 0.91, *p* < 0.0002.

**Table 1:** Validation of RNA-seq data using qPCR.

Gene ID	Description	RNA-seq	qPCR	RNA-seq	qPCR	RNA-seq	qPCR	RNA-seq	qPCR
		FC, 4 dpi		FC, 7 dpi		FC, 14 dpi		FC, 16 dpi	
106581616	*acod*	− 7.7	− 7.1	− 8.5	− 12.7	− 6.9	− 9	− 20.2	− 22.3
100136453	*cath1*	− 7.7	− 5.9	− 9.1	− 9.0	nd	**1.1**	− 2.5	− 2.5
100136446	*clra*	− 4.7	− 1.4	**34**	**75.9**	− 4.6	**1.1**	− 7.1	− 1.4
106585882	*cc4*	− 3.5	− 3.2	− 9.5	− 8.5	− 4.7	− 2.0	− 9.3	− 2.8
106577833	*il-8*	nd	**1.6**	− 3.2	− 1.3	nd	**1.8**	**5.2**	**2**
106600843	*il17a/f1*	− 11.3	− 12.2	− 10.8	− 9.6	− 2.5	− 3.2	− 10.9	− 8
106611589	*lect2*	− 3.1	− 2.3	**36.8**	**45.9**	− 3.7	− 2.2	− 6.9	− 5.8
100136358	*nos2*	− 31.2	− 17.8	− 54.3	− 50	− 11.7	− 6.8	− 35.1	− 23.9
106581433	*ptx3*	nd	− 1.2	− 1.4	− 1.7	− 1.4	− 2.1	− 2	− 2.5
106604759	*sap*	**2.2**	**2.5**	− 1.4	**1.1**	**1.7**	**2.9**	**1.6**	**2.5**
Spearman correlation		ρ = 0.91, *p* < 0.0002		ρ = 0.89, *p* < 0.0003		ρ = 0.78, *p* < 0.0008		ρ = 0.91, *p* < 0.0002	

The expression of ten genes was analysed by qPCR across all experimental time points. Genes included *acod1: aconitate decarboxylase 1*, *cath1: cathelicidin 1*, *clra*: *c type lectin receptor a*, *cc4*: *c–c motif chemokine 4-like*, *lect2: leukocyte cell-derived chemotaxin 2-like*, *il-8:interleukin-8-like *, *il17a/f1*: *interleukin*-*17a/f1-like* , *nos: nitric oxide synthase 2, inducible*, *ptx: pentraxin-related protein-like*, *sap: serum amyloid P-component-like*. nd: not detected, FC; fold change, dpi: days post infection. The RNA-seq and qPCR data were analysed using Spearman's rank order correlation. The correlation coefficient rho (ρ) range is − 1 to 0 to + 1, where 0 represents no correlation, − 1 a negative correlation and + 1 a positive correlation, *p* is the statistical significance. Up-regulated genes are highlighted (bold).

### Gene ontology (GO)

**Figure 4:**
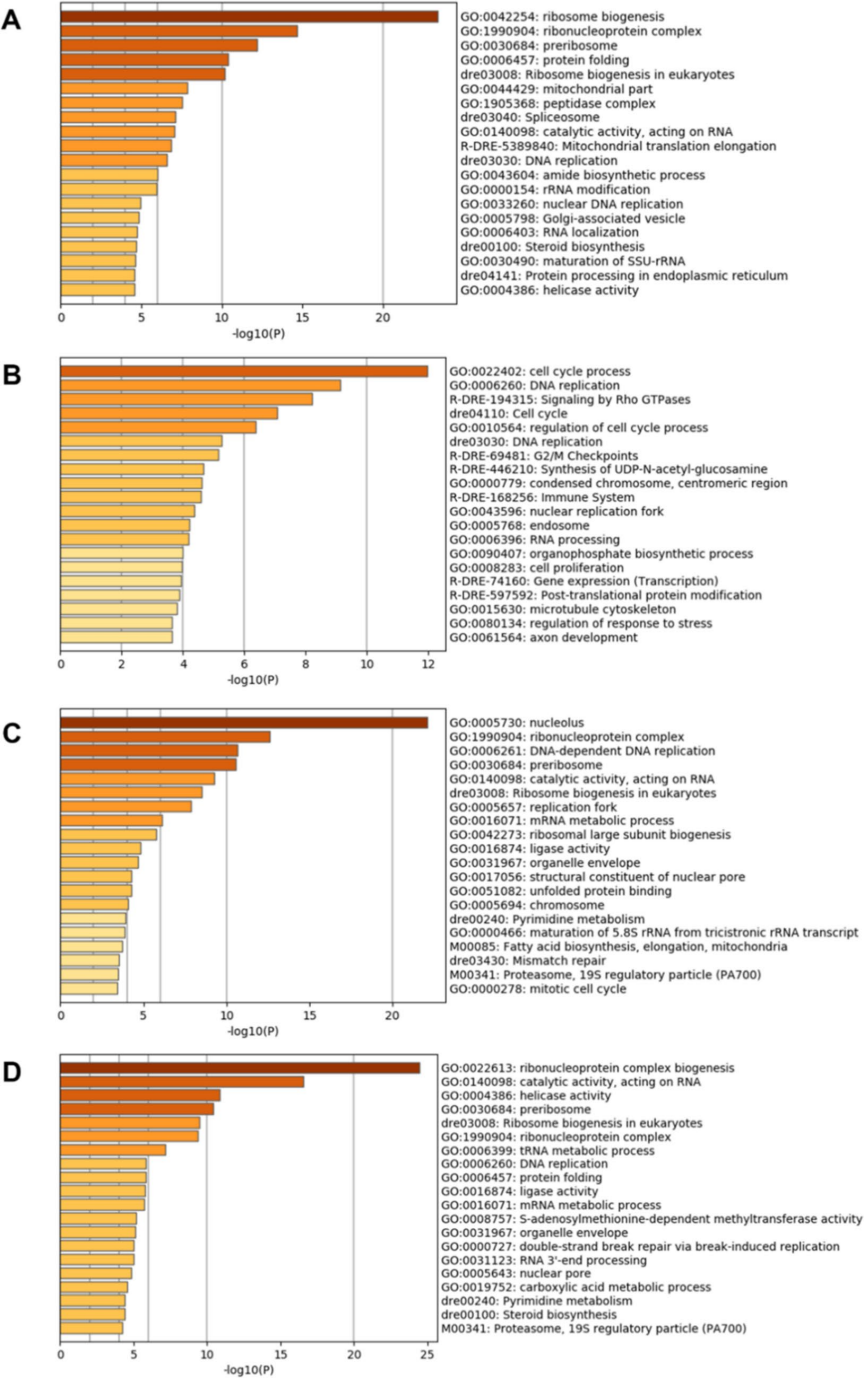
Top 20 ontology clusters enriched with down-regulated genes. (**A**) 4 dpi, (**B**) 7 dpi, (**C**) 14 dpi and (**D**) 16 dpi. The x-axis is the *p*-value in negative log base 10. The darker the colour of the horizontal column, the more significant the cluster enrichment. *Danio rerio* (dre) was used as the fish model species in Metscape^24^, where the naming convention for KEGG pathway is dre, and the reactome gene set is R-DRE.

**Figure 5:**
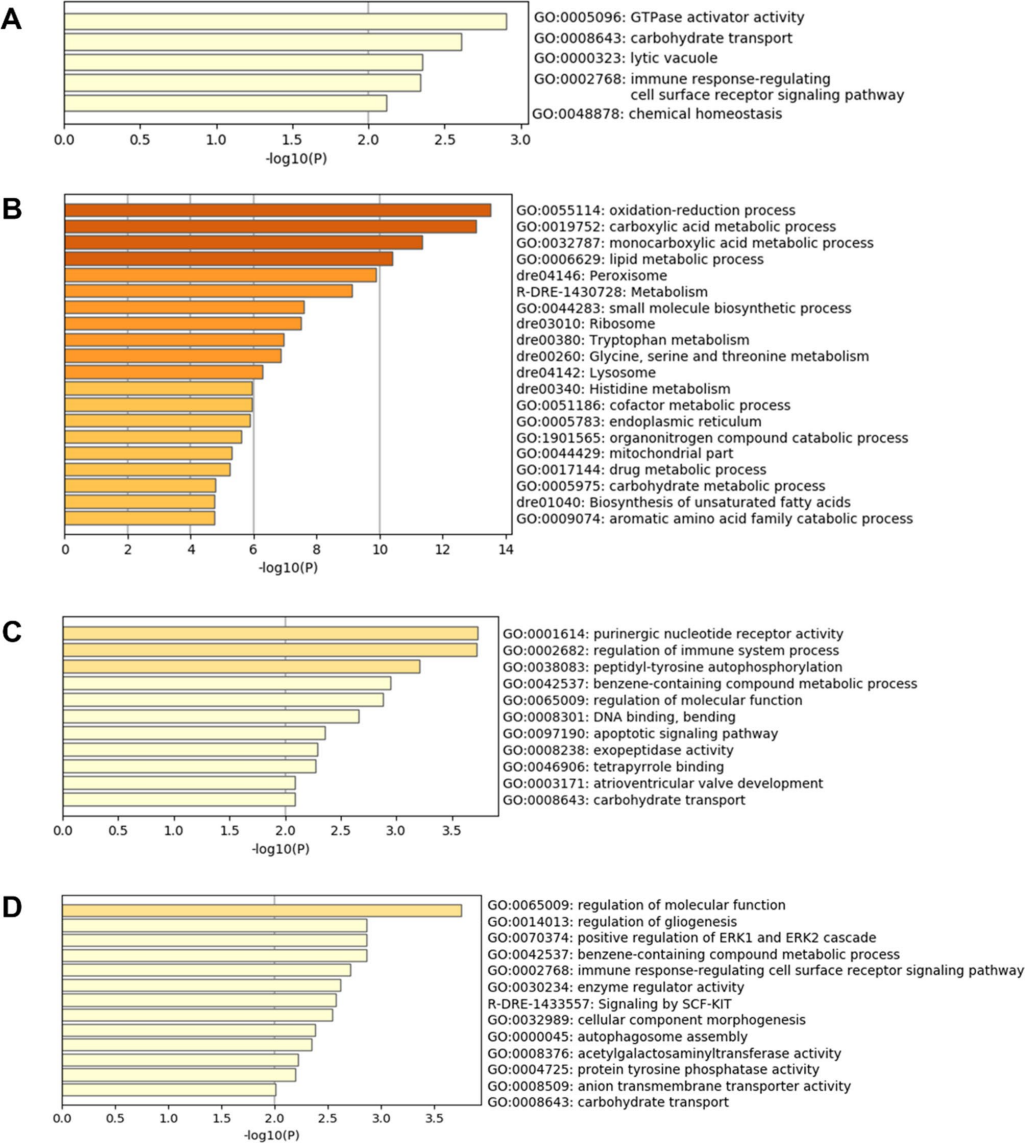
Ontology clusters enriched with up-regulated genes. (**A**) 4 dpi: 5 clusters, (**B**) 7 dpi: > 20 clusters, (**C**) 14 dpi: 11 clusters and (**D**) 16 dpi: 13 clusters. The x-axis is the *p*-value in negative log base 10. The darker the colour of the horizontal column, the more significant the cluster enrichment. *Danio rerio* (dre) was used as the fish model species in Metscape^24^, where the naming convention for KEGG pathway is dre, and the reactome gene set is R-DRE.

GO and pathway enrichment analyses were undertaken to identify the function of genes whose expression was differentially expressed in the gill following exposure to *P. perurans*. DEGs mapped to 224 Gene Ontology (GO) terms of which 140 were categorised as a biological process (BP), 45 as cellular components (CC), and 39 as having a molecular function (MF). Multiple BP terms were identified for epithelial cell migration, RNA processing, positive regulation of protein catabolic process, canonical Wnt signaling, regulation of Type 1 interferon, and the ERK1/ERK2 cascade. The negative regulation of NF-κβ (GO: 0032088) was also identified. Enriched ontology clusters were generated in Metscape^24^ at each time point (4, 7, 14 and 16 dpi) for terms associated with both down-regulated and up-regulated genes. In the top 20 ontology clusters enriched with down-regulated genes (Fig. 4), the reactone gene set R-DRE: 168256: Immune system was identified at 7 dpi (Fig. 4B) while in the top 20 ontology clusters enriched with up-regulated genes, GO: 0002768: immune response-regulating cell surface receptor signalling pathway was identified at 4 dpi (Fig. 5A) and 16 dpi (Fig. 5D), with GO: 0002682: Regulation of immune system process identified at 14 dpi (Fig. 5C). The oxidation–reduction biological process (GO: 0055114, − log10(*P*) 13.82) was identified as being the most enriched for up-regulated genes (108 genes) (Fig. 5B).

The top 20 down-regulated genes and the top 20 up-regulated genes at each for the 4 time points (4 dpi, 7 dpi, 14 dpi and 16 dpi) can be found in Supplementary Table S2 online and Supplementary Table S3 online, respectively.

### Gene down-regulation

At 7 dpi pathway and process enrichment analysis identified R-DRE-168256: immune system as being enriched with 51 down-regulated genes (Fig. 4B, Supplementary Table S4 online) genes, while PPI also identified this reactome as being enriched with 77 down-regulated genes. Further analysis of these 77 identified genes with having involvement in KEGG signaling pathways including the NOD-like receptor (NLR) signaling (dre04621, FDR 5.68e−08 *hsp90AB1, ikbkb, irf9, mapk3, nlrx1, sugt1, tbk1, stat1a, cybb*), Toll-like receptor (TLR) signaling (dre04620, FDR 1.5e−04, *tlr9, mapk3, tbk1, stat1a)* and RIG-1-like receptor signaling (dre04622, FDR 3.2e−04, *nlrx1, tbk1, mapk3k1, ikbkb*). In addition to NOD, TLR and RIG-1 pattern recognition receptors (PRRs), other members of this family include C-type lectin-like receptors (CLR) and cytosolic DNA sensors^25^. PRRs respond to pathogen-associated molecular patterns (PAMPs) or host-derived damage-associated molecular patterns (DAMPs) by triggering activation of NF-κβ, AP1, CREB, c/EBP, and IRF transcription factors^25^. In the current study, the *clra* gene (GeneID 100136446) was substantially up-regulated at 7 dpi (Table 1), which would suggest that the NLR, TLR and RIG-1 pathway is regulated differently as the genes associated with these pathways are down-regulated at 7 dpi. The Herpes simplex infection pathway (dre05168: FDR 0.00015) was also identified as being enriched with down-regulated genes *(ikbkb, irf9, mapk3, stat1a, tap1, tbk1, tlr9*). The PPI also identified reactome gene sets enriched with down-regulated genes from R-DRE-168256: immune system, the most significant of these being R-DRE-1280215: Cytokine signalling in the immune system, containing 15 genes (FDR 2.8e−15, *csf3r, dlg1, epgn, il-34, irf9, jak3, mapk3, nrg1, spred2b, stat4, syk, tbk1, tnfrsf11a, tnip2, ube2na*) of which all the genes with the exception of 2 genes (*tnfrsf11a and tnip2)* were also associated with R-DRE-449147: Signalling by interleukins (Supplementary Table S4 online).

### Cytokine gene expression

Interrogation of the DEG data set for interleukin (*il*) gene in AGD-affected gill identified multiple transcripts from nine *il* gene families whose expression was differentially expressed (*Il-1β*, *Il-8, il-11, il-12 β, il-17 (a/d/f) il-18, il-27β*, *il-3)* (Table 2). Interestingly, *il-17a/f1-like* (Gene ID 106600843) was down-regulated at every time point. Two interleukins were found to be up-regulated,: *il-27β* (Gene ID 106574502) was 12.4 log_2_FC at 7 dpi, and one of the 2 paralogues of *il-8* (Gene ID 106577833) had a 2.4 log_2_FC at 16 dpi.

**Table 2:** Relative gene expression of Interleukins in AGD-affected gill.

Time	GeneID	Description	Chr	p-adj	Log_2_FC
4 dpi	106570815	*il-1 beta-like*	ssa15	0.0008	− 1.6
	100136449	*il-1 beta*	ssa24	0.0221	− 0.9
	106601367	*il-17A-like*	ssa01	0.0016	− 1.1
	106600843	*il-17A/F1-like*	ssa01	0.0000	− 3.5
	106571057	*il-17F-like*	ssa15	0.0000	− 2.5
7 dpi	106570815	*il-1 beta-like*	ssa15	0.0005	− 2.6
	106562915	*il-1 beta-like*	ssa11	0.0235	− 1.2
	100136449	*il-1 beta*	ssa24	0.0014	− 2
	100195681	*il-8*	ssa06	0.0037	− 1.7
	106575827	*il-11*	ssa02	0.0061	− 2.3
	106603888	*il-12 subunit beta-like*	ssa04	0.0098	− 1.9
	106612126	*il-12 subunit beta-like*	ssa09	0.0203	− 1.6
	101448031	*il-15*	ssa04	0.0069	− 1.2
	106600843	*il-17A/F1-like*	ssa01	0.0000	− 3.4
	106571057	*il-17F-like*	ssa15	0.0010	− 2.4
	100196379	*Il-18*	ssa20	0.0041	− 1.4
	106574502	***il-27 subunit beta-like***	ssa16	0.0000	**12.4**
	106560860	*il-34*	ssa10	0.0426	− 0.7
	106573710	*il-34-like*	ssa16	0.0402	− 1.3
14 dpi	106600843	*il-17A/F1-like*	ssa01	0.0053	− 1.3
16 dpi	100136449	*il-1 beta*	ssa24	0.0075	− 0.9
	106562915	*il-1 beta-like*	ssa11	0.0103	− 0.8
	106570815	*il-1 beta-like*	ssa15	0.0009	− 1.4
	**106577833**	***il-8-like***	**ssa18**	**0.0210**	**2.4**
	100195681	*Il-8*	ssa06	0.0039	− 1
	106612126	*il-12 subunit beta-like*	ssa09	0.0069	− 0.6
	**100195864**	***Il-17D***	**ssa21**	**0.0202**	**0.6**
	106571057	*il-17F-like*	ssa15	0.0184	− 0.8
	106600843	*il-17A/F1-like*	ssa01	0.0000	− 3.4

GeneID: *Salmo salar* gene identification number, chr: chromosome location, p-adj: *P*-values adjusted for false discovery rate, Log_2_FC: log of gene expression fold change to base 2, up-regulated genes are highlighted with bold text, dpi: days post inoculation.

The DEG lists were further screened for additional cytokines including interferons (*ifn*) (Supplementary Table S5 online), tumour necrosis factor-alpha (*tnf-α*) and transforming growth factor-β (*tgf-β*) (Supplementary Table S6 online). With regard to interferon, only one gene was found in the data set, *ifn*α3 which was down-regulated at 16 dpi (− 1.3 log_2_FC). Alpha/beta receptor 1a-like and gamma receptors (*ifngr1a, 1b, 2b*) were found to be down regulated at the earlier time points of 4 and 7 dpi while numerous interferon regulatory factors (*irf*) were down-regulated throughout the study (*irf1, irf1 isoform 2, irf2a irf3, irf4-like, irf5, irf6-like, irf2a, irf7b, irf8-like, irf9). irf2-like (*GeneID 106612476*)* was up-regulated at 7 dpi and 14 dpi, while two paralogues of *irf4-like (*GeneID 106599909, 106571913*)* were up-regulated at 14 dpi (Supplementary Table S5 online). Down-regulation of *tnf-α (*− 3 log_2_FC) was seen at 7 dpi with *tnf-α1 precursor* (Gene ID 100136457) persistently down-regulated from 4 to 14 dpi (− 1.4 log_2_FC ± 0.2) and the *tnf-α2 precursor* (Gene ID 100136458) down-regulated at the earlier times of 4 dpi and 7 dpi. Upregulation of *tgf-β induced* and *tgf-β3-like* was evident at 7 dpi. One *tgf-β2-like* (Gene ID 106584161) was up-regulated at both 7 dpi and 16 dpi, while *tgf-β1-like* (Gene ID 106569003) was up-regulated at both 14 dpi and 16 dpi (Supplementary Table S6 online).

### Mucin gene expression

As AGD leads to the production of excessive mucus on the gills, the DEGs dataset was also interrogated for the expression of additional mucin gene expression (Supplementary Table S7 online). Mucins identified in the current study included * muc-2-like, 5AC-like, 7-like, 12-like 13-like, 17-like* and an *intestinal mucin-like* gene*,* with a possible similarity to *muc-2.* Interestingly, there were 5 paralogues of *muc-2-like* identified in this study at the earlier times of 4 dpi and 7 dpi, where all but one (Gene ID 106584523) was up-regulated (log_2_FC 3.0). Two paralogues of *muc-5AC-like* were present, with the expression of one (GeneID1 106612949) at 4 dpi up-regulated, while the other paralogue (GeneID1 106577588) was present at 7 dpi where the expression was down-regulated. One gene for *muc-7-like* was consistently down-regulated at every time point. The expression of *muc-12-like* was up-regulated (log_2_FC 0.9) at 14 dpi, while the expression of a *muc-13-like* (GeneID 106579100) was substantially up-regulated (log_2_FC 7.8) at 7 dpi.

### Immune gene up-regulation

There were 14 immune-related genes up-regulated at times other than 7 dpi associated with various biological processes (Table 3), including GO: 0002682: Regulation of immune system process *(btk, c1cq, CD79b, CD99, cgas, hmgb1a, hmgb3b, Il-34, kita, lyn, tfpi1, themis*), GO: 0002253: Activation of immune system process (*btk, c1cq, CD79b, cgas, hmgb1a, themis*), GO: 0097190: B-cell differentiation (*cd79b, Ikzf1.7, kita*), GO: 0002768: immune response-regulating cell surface receptor signalling pathway (*cd79b, kita, themis2*). Genes were also associated with the Reactome gene set R-DRE-983695: Antigen activates B-cell receptor (BCR) leading to generation of second messengers (*btk, cd79b, dapp*). Immune activation was evident from 7 dpi with complement activation (DRE-166658, FDR 0.0178) involving ten genes (*crp, c6, c8a, c8b, c8g, c9, cpb2, hbl3, masp1, masp2*) and is consistent with what was found in the proteome analysis of AGD-affected gill in our laboratory^26^. The most up-regulated gene was mannan binding lectin serine peptidase 2 (*masp2*, 30.0 log_2_FC) gene ID 106572058). Screening of the top 100 genes identified thirteen other complement transcripts also up-regulated including (*C1q-like protein 2* (× 4), *C1q-like protein 3, C2-like, C3-like* (× 2), *C9, factor B, factor H, properdin-like* (× 2). Also up-regulated at 7 dpi were genes involved in the acute phase response, an early innate immune function that is initiated by inflammatory signals, leading to the release of acute phase proteins into the bloodstream to re-establish homeostasis following microbial infection^27^. Genes involved in the acute phase response (APR) markedly up-regulated at 7 dpi in the AGD affected gill included *pentraxin, alpha-1-antitrypsin*, *alpha-2-macroglobulin*, *fibrinogen* (*α, β, γ*), *leukocyte cell-derived chemotaxin-2* (*lect2*), *C-type lectin receptor* (*clra*), and *serum amyloid P-component* (*sap*) (data not shown).

**Table 3:** Genes up-regulated in immune regulation and activation at 4, 14 and 16 dpi, but not at 7 dpi, in AGD-affected gill.

Gene ID	Gene symbol	Biological process and pathway	dpi
568653	*btk*	GO: 0002682, GO: 0002253, R-DRE-983695	14, 16
449803	*c1qc*	GO: 0002682, GO: 0002253	14, 16
100329481	*cd79b*	GO: 0002682, GO: 0002253 GO: 0097190, GO: 0002768, R-DRE-983695	4, 14, 16
559896	*cd99*	GO: 0002682	14
557043	*cgas*	GO: 0002682, GO: 0002253	14, 16
550386	*dapp1*	R-DRE-983695	14, 16
321622	*hmgb1a*	GO: 0002682 GO: 0002253	14, 16
550466	*hmgb3b*	GO: 0002682	14
560193	*Il-34*	GO: 0002682	14
30256	*kita*	GO: 0002682 GO: 0097190 GO: 0002768	4, 14, 16
30177	*ikzf1*	GO: 0097190	14, 16
447804	*lyn*	GO: 0002682	14
560339	*tfpi2*	GO: 0002682	14
100535600	*themis2*	GO: 0002682 GO: 0002253 GO: 0002768	4, 14, 16

Gene symbols: *btk: bruton agammaglobulinemia tyrosine kinase, c1qc: complement component 1, q subcomponent, C chain, cd79b: cd79b molecule, immunoglobulin-associated beta, cgas: cyclic GMP-AMP synthase, hmgb: high mobility group box, il-34: interleukin-34, kita: kit proto-oncogene, receptor tyrosine kinase a, ikzf1:IKAROS family zinc finger 1, lyn:lyn proto-oncogene, Src family tyrosine kinase, tfpi2: tissue factor pathway inhibitor 2, themis2: thymocyte selection associated family member 2.* Biological processes: GO: 0002682: Regulation of immune system process, GO: 0002253: Activation of immune system process, GO: 0097190: B-cell differentiation, GO: 0002768: immune response-regulating cell surface receptor signalling pathway. Reactome gene set: R-DRE-983695: Antigen activates B-cell receptor (BCR) leading to generation of second messengers. dpi: days post inoculation.

## Discussion

Despite the growing number of gene expression and proteomic studies being carried out on AGD-affected salmon (reviewed in Marcos-Lopez & Rodger 2020)^4^, there are still knowledge gaps remaining regarding host immune response in early stage disease. The progression of AGD on the gill can be monitored visually and scored based on the absence or presence of mucoid patches and their distribution on the gills^6^. In the current study, AGD mucoid lesions were not evident on the gills in AGD-affected groups at the earlier time points (4 and 7 dpi) and the gill score in the AGD-affected fish remained less than 2 by day 16. Although it is known that long term cultivation of *P. perurans* results in the loss of parasitic virulence *in vivo*^28^*,* the amoeba used in the current study were twelve weeks in culture prior to use in the challenge trial. The progression of the disease based on gill scores observed raised a concern in relation to the virulence of the pathogen. However, at the first sampling point (4 dpi) *P. perurans* DNA was detected by qPCR and microscopically, there was evidence of epithelial hyperplasia. Amoebic virulence was therefore shown to be adequate and the rate of disease progression appropriate in order to investigate gene expression at early disease stages. Moreover, at 4 dpi, 4838 genes were differentially regulated with 2985 genes down-regulated and 1853 up-regulated. By 7 dpi the pattern of gene expression changed, with more genes up-regulated and to a greater extent (Fig. 2). In addition to hyperplasia, fusion of the lamellae was now evident, indicative of AGD in the absence of visible mucoid patches. At 14 dpi and 16 dpi, the pattern of gene expression returned to that which was seen at 4 dpi. Principal component analysis of the 7 dpi time point showed four of the six AGD-affected samples clustered together and were clearly different from the other fish sampled. With the other samples in the 7 dpi AGD-affected group, one sample clustered with the pre-7 dpi samples, while the other sample clustered with the post-7 dpi samples. The fish were exposed to the *P. perurans* by immersion. In comparison to intraperitoneal injection methods, the number of amoeba colonising the gill of each fish cannot be controlled, so this finding was not unexpected and highlights the importance of using individual instead of pooled samples for fish studies, and having a minimum of 6 fish per group for RNA-seq AGD studies^29^. For this study, the same gill (gill 2) was taken from each fish with no pre-selection for the presence or absence of mucoid lesions.

The RNA-seq data was validated using qPCR analysis of a panel of immune genes. Down regulated genes at each time point included *acod1*, *cc4*, *il-17a/f1* and *nos2*, and *ptx3* with *cath1* being down-regulated at 4, 7 and 16 dpi. *Acod1*, also known as immune responsive gene 1 (*irg1*) is a gene coding for an enzyme producing itaconic acid through the decarboxylation of cis-aconitate. The expression levels of *acod1/irg1* correlates to itaconic acid production, an indicator of inflammation activation in mammals^30^. The *acod1* gene is emerging as a regulator of immuno-metabolism in inflammation and infection^31^. In the current study, the pattern of expression of *acod1/irg1 and nos2* were similar with both genes being consistently down-regulated. Furthermore, both *nos2* and *acod1* genes were also shown to have involvement in the biological process ‘GO: 0072593: Reactive oxygen species metabolic process’ containing 7 down-regulated genes (*nos1, ncf1*, *cybb, nos2a, noxa1, hace1, acod1*) at 7 dpi. The lack of an increase in *nos2* expression in the current study, is consistent with the previous finding that there was no detectable increase in the expression of *iNos (nos2)* between healthy and AGD-affected fish during early disease stage^15^. In a previous study, the differential expression of the superoxide dismutase (*sod*) and catalase (*cat*), two genes involved in oxidative stress, were reported to be up-regulated in late stage AGD^20^. SOD catalyses the dismutation of the superoxide (O_2_^−^) into O_2_ or H_2_O_2_ while CAT plays a role in protecting cells from oxidative damage. In the current study, at 7 dpi there were 108 genes identified as being up-regulated in GO: 0055114, the oxidation–reduction biological process (Fig. 5B). Three *sod* genes were found to be up-regulated including soluble *sod1*, (GeneID: 30553), the mitochondrial *sod2* (GeneID: 335799) and the extracellular *sod3b* (GeneID: 794006). In the same study, *cat* gene expression was reported as being significantly upregulated in gill lesion tissue with gill score 2. In the current study, catalase was not found to be up-regulated prior to the appearance of lesions on the gill, at 7 dpi, further confirming this gene as a possible lesion specific marker for AGD. The up-regulation of genes involved in the oxidative-reduction biological process during early stage AGD will be further investigated in a future study. TGF-β1 is a potent suppressor of *nos2* by multiple mechanisms in numerous cell types, including macrophages^32^. An up-regulation of *tgf-β* isoforms (*β1, β 2, induced*) was evident in the current study from 7 dpi. TGF-β can promote IL-17 cell differentiation however the expression of *Il-17a/f1* was consistently down-regulated. CC4 is a chemokine for natural killer cells, monocytes and a variety of other immune cells^33^. LECT2 is a chemotactic factor attracting neutrophils to the site of infection. The genes *lect2* and *clra* showed the same pattern of expression being substantially up-regulated at 7 dpi, while being down-regulated at the other time points. Indeed it is the interaction between LECT2 and C-type lectin receptor (CLRA) proteins that is thought to be responsible for the ‘‘neutrophil-chemotactic’’ characteristic of LECT2^34^. Furthermore, *Lect2,* along with *ptx* and *sap* are acute phase response genes. Interestingly, the expressions of *ptx* and *sap* were not as expected. While *ptx* (GeneID 106581433) was found to be down regulated, and *sap* (GeneID 106604759) up-regulated approximately twofold, paralogues of *sap* (GeneID 106608633) and *ptx* (GeneID 100136583) were both substantially up-regulated at 7 dpi. Previous findings reported no differential gene expression of an SAP pentraxin in AGD-affected gill having visible mucoid lesions^13^. It is noteworthy that *Il-8,* also a chemotactic factor with similar functions to *lect2,* did not show a similar expression trend, and was down-regulated at 7 dpi, and up-regulated at 16 dpi.

From the RNA-seq data set, down-regulated immune genes at 7 dpi were identified in the Reactome gene set R-DRE-68256: Immune system, which were associated with NOD-like receptor (NLR), Toll-like receptor (TLR) and RIG-1 like receptor signaling pathways, and also cytokine and interleukin signalling. NOD-like receptors (NLRs) can initiate or regulate host defence pathways through formation of signalling platforms that subsequently trigger the activation of inflammatory caspases and NF-κβ^35^. A recent study of advanced stage AGD (day 21, gill score 3.3) reported activation of such defence pathways, with upregulation of genes mapped to NLR, TLR signalling, and Herpes simplex virus 1 infection pathways^36^. The current study also identified NLR and TLR signalling pathways, but enriched for down-regulated genes at 7 dpi. Involvement of the Herpes simplex virus 1 infection pathway suggests a viral-like response to parasite invasion in AGD^36^. In the current study the Herpes simplex infection pathway (dre05168: FDR 0.00015) was also enriched with down-regulated genes at 7 dpi, suggesting that the anti-viral response is either not activated, or suppressed, in early stage AGD, a theory that is further supported by down-regulation of genes associated with the biological process GO: 0060337: Type 1 interferon signaling pathway, and interferon regulatory factors. Other pathways enriched with down-regulated genes at 7 dpi included B-cell receptor (BCR) signalling, T-cell receptor (TCR) signalling*,* the differentiation of Th1/Th2 cells, and Th17 cells. Interestingly, these signalling pathways had 2 genes in common*, ikbkb* and *mapk3.* Host invasion by pathogens frequently induces activation  of NF-κβ, which plays an important role in initiation of innate immune responses by regulating the expression of many immunological mediators, including chemokines, cytokines, adhesion molecules, and enzymes that produce secondary inflammatory mediators^37^. Based on the pattern of gene expression in the current study, specifically at 7 dpi, *P. perurans* may have developed an immune evasion strategy to prevent the activation of NF-κβ during the early onset of AGD. Of particular interest is the down regulation of genes involved in NOD-like receptor pathway, perpetuating the inhibition of NF-κβ by Iκβ. Pathogens have previously been reported to have developed strategies to circumvent the activation of the NF-κβ activation, by preventing the inhibitor, Iκβ, from being ubiquitinated and therefore preventing its degradation, causing NF-κβ to remain sequestered in the cell cytoplasm and therefore inactive^38^. Indeed, some viruses for example vaccinia viral protein B14, encode virulence factors, to target IKKβ to inhibit the NF-κβ-mediated antiviral immune response^39^. Therefore it is conceivable that *P. perurans* virulence factors could potentially have some immunomodulatory effects on their host.

With regard to the down-regulation of genes with involvement in cytokine and interleukin signalling, interrogation of the DEG dataset identified multiple transcripts from nine interleukin (*il*) gene families with differential expression in AGD-affected gills, with one gene *il-17a/f1-like* (Gene ID 106600843) was found to be down-regulated at every time point. Only two interleukins were found to be substantially up-regulated, *il-27β* at 7 dpi, (12.4 log_2_FC), and one of 2 paralogues of *il-8* (Gene ID 106577833) at 16 dpi (2.4 Log_2_FC). The up-regulation of *Il-1β* has been reported as the hallmark of late stage AGD infection^17^ associated with larger AGD-lesions. In these cases the observed increase in mucous cell hyperplasia has led to the contention that mucous cells are the potential source of *il-1β*^13^. Multiple paralogues *of il-1β* were identified in the current study with down-regulated expression seen at 4 dpi, 7 dpi and 16 dpi, suggestive of an impaired inflammatory response. Two paralogues of *Il-12β-like,* the p40 subunit for IL-12 and IL-23 were also down-regulated at 7 dpi. IL-12 is a growth factor for Th1^40^ while IL-23 is involved in Th17 cell differentiation^41^. IL-17 is a key cytokine produced by Th17 cells, involved in the inflammatory and neutrophil response. IL-17A is produced mainly in T cells, whereas IL-17F is produced in T cells, innate immune cells, and epithelial cells^42^. The role of IL-17F is mainly in the mucosal host defence mechanisms^43^. A recent study reported the expression of *Il-17a/f1b* and *Il-17d* to be significantly down-regulated in comparison to the negative control in gills from fish inoculated with a high concentration of *P. perurans* trophozoites (5000 amoeba /L)^12^. In the current study where fish were infected with 2750 amoeba/L, *Il-17a-like* was also found to be down-regulated at 4 dpi, while *il-17a/f1* was consistently down-regulated at every time point. Interleukins involved in inducing IFN-γ, which promotes Th1 cell differentiation, *Il-15* and *Il-18,* were also down-regulated in the current study. Interestingly, the expression of *Il-27β*, a component of both interleukins IL-27 and IL-35, was substantially up-regulated at 7 dpi (12.4 log_2_FC). IL-27 is unique in that although it induces Th1 differentiation, it can antagonise the development of the Th17-cell response and limit Th-17 driven inflammation^44^, critical for host defence against bacterial, fungal and viral infections at mucosal surfaces^40^ while IL-35 induces proliferation of Treg cell populations but reduces activity of Th17 cell populations^45^. In addition to the down-regulation of interleukins, numerous *ifn* receptors and regulatory factors were down-regulated throughout the study, with the exception of *irf2-like and irf4-like* which were both up-regulated at 14 dpi. The down-regulation of *tnf-α was* also evident.

Mucins are high molecular weight glycoproteins secreted by goblet cells and the main structural component of mucus. Excessive mucus production in the gills is a hallmark of AGD^3^ with substantial up-regulation of the secreted MUC5AC detected in clinical AGD^14^. Specialized epithelial (goblet) cells are the major source of MUC5AC, which can be induced by MMP9 through the activation of the epidermal growth factor receptor (EGFR) and mitogen-activated protein kinase 3/2 MAPK 3/2(ERK1/2) cascade^46^. The expression of three genes (*kita, hmgb1a, gpr183a*) involved in the positive regulation of the ERK1/2 cascade (GO: 0070374) were found to be upregulated at the last time point (16 dpi) in this study. Mucins (muc) identified in the current study included *muc-2-like, 5AC-like, 7-like, 12-like 13-like, 17-like* and an *intestinal mucin-like* gene (Supplementary Table S7 online). Two paralogues of *muc-5AC-like* were found to be differentially expressed, with one paralogue at 4 dpi up-regulated, while the other paralogue at 7 dpi was down-regulated. Interestingly, the expression of a *muc-13-like* was found to be substantially up-regulated (log_2_FC 7.8) at 7 dpi. Muc-13 is associated with mucosal immunity, and is an epithelial and hemopoietic cell surface mucin that protects against inflammation and may also play a role in cell signaling^47,48^. Muc-13 has also been reported as a quantifiable host marker of plasmodium parasite infection which could potentially be used to distinguish infected from uninfected cells^49^. A recent RNA-seq study also reported the differential expression of a mucin-13-like gene in the head-kidney of advanced stage AGD-affected fish but not the gill^36^. Further investigation is warranted to determine the role of *muc-13-like* in the various tissues of AGD-affected fish and if the expression of this mucin is triggered through *P. perurans* cell attachment.

The present study provides the initial discovery and description of cytokine down-regulation during the amoeba attachment phase of  early-stage AGD in the gill of Atlantic salmon, and provides the basis for future, more in-depth studies to elucidate the mechanisms behind this potential immune evasion strategy.

## Methods

### Study design

This early stage AGD study was designed to compare the transcriptomic profile from individual gill tissue from 6 fish at 4 time points (4, 7, 14 and 16 dpi) to 6 uninfected control fish (0 dpi). As the 0 dpi time point was critical, it was decided to include two groups of 6 control fish (T0_GCRL, T0_GAGD) in the RNA sequencing. As all six fish in both control groups had a similar gene profile, the T0_GCRL group was subsequently selected for the data analysis. The experiment was designed to establish an AGD challenge with a type I error of 5% assuming a success rate of 80% (power analysis) and also taking into consideration a recent paper on biological replicates for RNA-seq^29^.

### Fish husbandry

Atlantic salmon smolts (78.8 ± 20.6 g) were sourced from a local freshwater hatchery on the west coast of Ireland and transported to the Marine and Freshwater Research Center, at the Galway Mayo Institute of Technology. Fish are were distributed into 8 circular black 1 m^−3^ L tanks (45 fish/tank) connected to recirculating aquaculture systems at a stocking density of 3.6 kg m^−3^, water temperature 12 °C, artificial seawater (30ppt) and a 12 h/12 h light/dark cycle. Fish were acclimatized for 6 days prior to the start of the trial. Fish were fed a commercial salmon diet (Le Gouessant) at 1% body weight per day. The *in-vivo* fish trial was carried out according to the ARRIVE guidelines for animal research^50^. This project was authorised by the Health Products Regulatory Authority (HPRA), authorisation number AE19137/P001, in compliance with Directive 2010/ 63/EU transposed into Irish law by S.I. No 543 of 2012.

### *Paramoeba perurans* isolation and culture

*Paramoeba perurans* trophozoites were collected by gill swabbing from AGD infected Atlantic salmon on a commercial salmon farm on the west coast of Ireland. Amoebae were cultured on marine yeast agar plates (MYA; 0.01% malt, 0.01% yeast, 2% Bacto Agar), 16 °C overlaid with 7 ml sterile sea water^51^, and sub-cultured weekly by transferring free-floating cells to fresh MYA plates. Confirmation of *P. perurans* identity was performed using qPCR as previously described by Downes (2015)^5^. The amoeba was in culture for 12 weeks prior to challenge.

### *Paramoeba perurans* challenge

Six MYA agar plates were scraped gently and the sterile seawater (SSW) overlay and cells collected into a 2 × 50 ml tubes. The volume of each tube was brought up to 40 ml using SSW at 16 °C, centrifuged at 800×*g* for 10 min at 16 °C. The supernatant, with the exception of the last 5 ml was removed from each tube. The pellet was re-suspended in the remaining 5 ml and then both 5 ml were combined into one 10 ml concentrate of amoeba. In a 15 ml tube, a 1:40 dilution was made using 250 µl of amoeba into 9.75 ml SSW. The tube was closed and inverted several time to ensure proper distribution of the amoeba. The diluted amoeba (1 ml) were placed on a Sedgewick rafter counter chamber. The cells were allowed to settle on the chamber for 10 min prior to counting. Counts were performed in triplicate. After an acclimatisation period of 6 days, 90 fish were challenged with *P. perurans (*2750 amoebae/L) in 300 L for 4 h in artificial seawater (30ppt),  with oxygen saturation, water temperature (12 °C)*,* and fish behaviour and welfare closely monitored. The 90 control fish were also held at 300 L for 4 h under the same conditions without *P. perurans.* Following the challenge, fish were returned to their respective tanks to be held under the same conditions as prior to challenge.

### Disease progression

Clinical symptoms of AGD were determined by macroscopic examination of the intact gills in euthanised fish and were scored according to Taylor et al.^6^. Prior to sampling, 3 fish were taken from each duplicate control and AGD-affected tanks, and all 6 fish placed in an anaesthetic bath. Naïve control fish (6) and experimental fish (6) were sampled at each time point (0, 4, 7, 14 and 16 dpi) and all fish were gill scored.

### Sample collection

The current study was carried out in parallel with another study investigating the proteomic expression in early stage AGD from fish (6 control, 6 AGD-treated) sampled at 9 time points over 16 days (0, 1, 2, 3, 4, 7, 9, 11, 14, 16 dpi)^26^. Prior to sampling, 3 fish were taken from each duplicate control and AGD-affected tanks, and all 6 fish placed in anaesthetic at the same time. Fish were euthanised by overdose of anaesthetic (400 mg L^−1^ tricaine methane sulfonate) and the gills examined and scored based on the absence (0) or presence (1 to 5) of the mucoid patches on euthanised fish^6^. Prior to the tissue sampling, the fish were bled from the caudal vein. To minimize blood contamination in gill tissue, prior to extraction, the gills were perfused by injection of 2 ml phosphate buffered saline (PBS) into the heart. For RNA sequencing the entire second left gill, irrespective of the presence of absence of mucoid lesions, was removed with the arch and placed in 300ul of RNA*later (*Ambion Inc, Austin, Texas), stored overnight at 4 °C before being transferred to − 80 °C. For histopathology, the first right gill with arch was fixed in 10% buffered neutral formalin solution prior to being routinely processed and embedded in paraffin wax blocks. Gill tissue sections (5 µm) were stained with haematoxylin and eosin (H&E), and examined using an Olympus BX41 Microscope and CellSens software (Olympus, Tokyo, Japan) and imaged.

For amoeba identification, the fourth right gill with the arch was removed from individual fish and collected into 300ul of RNA*later (*Ambion Inc, Austin, Texas) prior to DNA extraction (DNeasy Blood and Tissue Kit, Qiagen) from individual gills. qPCR analysis was performed using a Taqman assay developed for the detection of *P. perurans* DNA^5^.

### Total RNA extraction

Following the removal of the whole, 2nd left gill from RNALater, Total RNA was extracted using the RNeasy Mini Plus Kit (Qiagen, Germany) according to the manufacturer’s instructions. Briefly, 30 mg gill tissue from each individual fish gill, irrespective of the presence of absence of mucoid lesions, was homogenized using a bead mill (Fisher Scientific) and 2.8 mm ceramic beads in 350 µl RLT lysis buffer and 1 µl DX antifoam reagent (Qiagen, Germany), 5 pulses/sec, 10 s, repeated 3 times. The optional DNase I (Qiagen, Germany) step was included to ensure complete elimination of gill genomic DNA. RNA was eluted in 70 µL of nuclease -free water and stored at − 80 °C until required. RNA was quantified using the Qubit RNA Assay Kit in Qubit 2.0 Fluorimeter (Thermo Fisher Scientific, USA). RNA integrity (RIN) was assessed using the RNA Nano 6000 Assay Kit of the Bioanalyzer 2100 system (Agilent Technologies CA, USA). Total RNA with RIN ≥ 8.0 or higher were used for library construction.

### Library construction and transcriptome sequencing

Library construction and transcriptome sequencing was outsourced (Novogene, Cambridge, UK). Total RNA from 6 control fish (0 dpi) and 6 fish from 4, 7, 14 and 16 dpi dpi was used for the construction of 30 RNA sequencing libraries using NEBNext Ultra RNA Library Prep Kit for Illumina (NEB, USA) according to manufacturer’s instructions. Index codes were added to attribute sequences to each sample. The clustering of the index-coded samples was performed on a cBot Cluster Generation System using HiSeq PE Cluster Kit cBot-HS (Illumina) according to the manufacturer’s instructions. After cluster generation, library preparations were sequenced on an Illumina Hiseq 2000 platform and 125 bp/150 bp paired-end reads were generated. FastqQc (Version 0.11.8) (www.bioinformatics.babraham.ac.uk/projects/fastqc/) was utilised for quality assessment of reads from each sample and Multiqc (Version 1.7)^52^ was used to visualise all FastQc results. No samples were identified as being bad quality, and all samples were included in the next step of the workflow process. Trimmomatic (v0.36)^53^ was used to trim paired reads in FASTQ files, using default parameters for paired-end mode and a minimum read length of 50 bp.

### Differential expression analysis

Differential expression analysis was performed on total RNA from 6 fish from 4, 7, 14 and 16 dpi using the DESeq2 (Version 1.24.0)^54^ where each time point was compared to 6 fish from time 0, naïve, pre-AGD samples. DESeq2 provided statistical routines for determining differential expression in digital gene expression data using a model based on the negative binomial distribution. The resulting *p* values were adjusted to control for the false discovery rate (FDR)^55^. Genes with an adjusted *p*-value (p-adj) of < 0.05 were determined to be differentially expressed. No minimum log_2_FC threshold was assigned as a cut-off value for differential expression. Four lists of DEGs were generated: T0 vs T4 (4 dpi), T0 vs T5 (7 dpi), T0 vs T8 (14 dpi) and T0 vs T9 (16 dpi) down. Each list was further divided into down-regulated and up-regulated genes resulting in a total of eight DEGs lists. Volcano plots were created to visualise the DEGs at individual time points (Fig. 2).

### Read mapping to the Atlantic salmon (*Salmo salar*) reference genome

The *Salmo salar* genome ICSASG_v2 (GenBank:GCF_000233375.1)^23^ was used to map the reads (Supplementary Table S1 online). Mapping was implemented using HiSat2 (version 2.1.0)^56^ using default parameters and paired-end mode. Counts were generated using featureCounts (v1.6.0)^57^ using the default parameters for paired-end reads. RNA-seq specific QC, sample correlation and visualisation were implemented using Seqmonk (Version 1.45.1) (https://www.bioinformatics.babraham.ac.uk/projects/seqmonk/).

Principal component analysis (PCA) plots were generated to visualise the relationships between all samples, as well as samples by time point. The similarity between samples from thirty RNA-seq libraries (6 fish × 5 groups) was analysed (Supplementary Fig. S1 online). An additional T0 group (6 individual fish) was sent for sequencing as an added precaution as this time point was considered critical to the success of the study. The RNA sequencing from all six fish in both control groups (T0_Ctrl, T0_AGD) was similar with regard to the total number of reads per fish and also in the total number of reads mapped (T0 Ctrl: 58312820 with 88.42% mapped, T0 AGD: (pre-inoculation) 56224414 with 88.03% mapped). A pairwise comparison between T0_Ctrl and T0_AGD identified only 1 DE gene between the 2 groups. Subsequently, the T0_GCRL fish (n = 6) were selected for the pairwise analysis of the RNA-seq data to the other time points.

### Pathway and process enrichment analysis

Gene Ontology (GO)^58,59^ and Kyoto Encyclopedia of Genes and Genomes (KEGG)^60^ pathway mapping was performed using Metscape^24^. The zebrafish (*Danio rerio*) database was used to determine GO enrichment as the option to use *Salmo salar* was not available in Metscape. For each given gene list in the DEG dataset, the following ontology sources were used: KEGG Functional Sets, KEGG Pathway, GO Biological Processes, GO Cellular Components, GO Molecular Functions, KEGG Structural Complexes and Reactome Gene Sets. A user-supplied list of 5815 protein coding genes, identified as *D. rerio* homologs, was used as the enrichment background. Terms with a *p*-value < 0.01, a minimum count of 3, and an enrichment factor > 1.5 (the enrichment factor is the ratio between the observed counts and the counts expected by chance) were collected and grouped into clusters based on their membership similarities. More specifically, *p*-values were calculated based on the accumulative hypergeometric distribution^61^, and *q*-values were calculated using the Benjamin-Hochberg procedure to account for multiple testings^55^. Kappa scores^62^ were used as the similarity metric when performing hierarchical clustering on the enriched terms, and sub-trees with a similarity of > 0.3 were considered a cluster. The most statistically significant term within a cluster was chosen to represent the cluster.

### Protein–Protein interaction (PPI)

PPI was carried out using STRING (version 11.0, https://string-db.org) to generate protein association networks based on function for immune genes identified as enriched in KEGG pathways and Reactome gene sets. The default settings were used and *Danio rerio* selected as the species of interest as STRING does not provide *Salmo salar* as a species option.

### Validation of RNA-seq data using Real-time PCR

Total RNA (1 µg) from each individual fish gill sample at 5 time points (0 dpi, 4 dpi, 7 dpi, 14 dpi and 16 dpi) was reverse transcribed to complementary DNA (cDNA) using the GoScript kit (Promega) as per manufacturer’s instructions. Real-time PCR was performed using 48.48 Dynamic Array Integrated Fluidic Circuit (IFC) chips on the Biomark HD system (Fluidigm, USA). Ten genes differentially regulated in the gill RNA-seq data were selected including *acod1: aconitate decarboxylase 1* (also known as *irg1*), *cath1: cathelicidin 1*, *clra*: *c type lectin receptor a*, *cc4: c–c motif chemokine 4-like*, *lect2: leukocyte cell-derived chemotaxin 2-like*, *il-8: interleukin-8-like*, *il17a/f1:* *interleukin-17a/f1-like, nos: nitric oxide synthase 2, inducible*, *ptx: pentraxin-related protein-like*, *sap: serum amyloid P-component-like*. *Elongation factor 1 alpha* (*ef1a)* was selected as the most appropriate housekeeping gene^63^. Gene primers were designed using PrimerQuest (Integrated DNA Technologies, https://eu.idtdna.com/). All primers were designed to have Tm of 62 °C and to be run in the qPCR at 58 C. Primer sequence details are provided in Supplementary Table S8 online.

A pre-amplification multiplex step was carried out on the target genes using a MiniAmp Plus PCR machine (Applied Biosystems) using the Preamp Master Mix (Cat. No. 100-5581, Fluidigm) as per manufacturer’s instructions. The pre-amplified cDNA was treated with Exonuclease I to remove unincorporated primers prior to running on the IFC chip. The PCR assay mix consisted of 0.7 µL of 50 mM primer mix (IDT, Belgium), 3.5 µL of 2X assay loading reagent (Fluidigm) and 2.8 µL of 1X DNA elution buffer (Qiagen, Germany). The sample premix was prepared with 200 µL of 2X SsoFast EvaGreen supermix with low ROX (Bio-Rad, München, Germany) and 20 µL of 20X Binding Dye Sample Loading Reagent (GE 48.48 Dynamic Array™ Sample & Assay Loading Reagent Kit, Item 85000821, Fluidigm), of which 3.85 µL of the sample pre-mix was combined  with 3.15 µL of the diluted pre-amplified PCR product for each sample inlet of the IFC chip. The PCR program was 95 °C for 60 s, 30 cycles of 95 °C for 5 s, 58 °C for 20 s followed by a melt curve protocol of 55 °C to 95 °C with a ramp rate of 1 °C/3 s. Each sample was run in triplicate/chip and 3 chips used per validation. Melt curve analysis was preformed to confirm the specificity of the amplified PCR product. PCR amplification efficiency (E) was calculated for each gene of interest and the housekeeping gene by the generation of standard curves using tenfold serial dilutions of the cDNA template (standard curve: R^2^ > 0.980, amplification efficiency range 90–105%). Melt curve analysis was used to confirm the amplification of single, PCR products. The relative expression for each gene was calculated using the delta delta CT (ddCT) method^64^ by the Real-Time PCR Analysis software on the Biomark HD (Fluidigm, USA).

### Statistical analysis

For pathway and process enrichment analyses, *p*-values < 0.01 were calculated based on the accumulative hypergeometric distribution, and *q*-values calculated using the Benjamin-Hochberg correction for multiple testing using Metascape. Kappa scores were used as the similarity metric when performing hierarchical clustering on the enriched terms, and sub-trees with a similarity of > 0.3 were considered a cluster. The most statistically significant term within a cluster was chosen to represent the cluster. For qPCR analysis, the fold change of each gene at each time point was analysed relative to the T0 control using an un-paired t-test with differences considered significant at *p* < 0.05. Ggplot2_3.2.1 in R studio Version 3.5.1 was used to generate the Spearman correlation data.

## Supplementary Information

Supplementary Information.

## Data Availability

All data supporting this study are included in the results section, the Supplementary material section online and openly available from the NCBI Geo Database, Accession Number GSE179972.
