# Supplementary material for: Investigation of the transcriptomic response in Atlantic salmon (Salmo salar) gill exposed to Paramoeba perurans during early onset of disease
Source: Sci Rep. 2021 Oct 19;11:20682. doi: 10.1038/s41598-021-99996-1 (PMC8526816; doi:10.1038/s41598-021-99996-1)
Supplement: Supplementary file 1 — Supplementary Information. [file 41598_2021_99996_MOESM1_ESM.docx]

Investigation of the transcriptomic response in Atlantic salmon (*Salmo salar*) gill exposed to *Paramoeba perurans* during early onset of disease.

Anita Talbot^1*^, Laura Gargan^2^, Grainne Moran^1^, Louis Prudent^1^, Ian O’Connor^1^, Luca Mirimin^1^, Jens Carlsson^2^, Eugene MacCarthy^1^

Affiliation: Galway Mayo Institute of Technology^1^, University College Dublin^2^.

**Supplementary Tables**

**Supplementary Table S1.** Reads mapped to the Atlantic salmon (*Salmo salar)* reference genome ICSASG_v2 (GenBank:GCF_000233375.1). HiSat2 (version 2.1.0) was used for mapping using default parameters and paired-end mode. Counts were generated using featureCounts (v1.6.0) using the default parameters for paired-end reads.

| Sample | No. raw reads | No. clean paired reads | % clean paired reads | No. aligned fragments (HiSats) | % aligned (HiSat2) | No. assigned fragments (featureCounts) |
| --- | --- | --- | --- | --- | --- | --- |
| 0 dpi_F1 | 23,705,616 | 21,788,776 | 91.91 | 20,939,014 | 96.1 | 17,067,854 |
| 0 dpi_F2 | 31,513,874 | 29,303,994 | 92.99 | 28,339,893 | 96.71 | 23,108,152 |
| 0 dpi_F3 | 29,391,335 | 27,346,835 | 93.04 | 26,015,044 | 95.13 | 21,097,194 |
| 0 dpi_F4 | 31,983,472 | 30,118,436 | 94.17 | 29,115,492 | 96.67 | 23,450,006 |
| 0 dpi_F5 | 27,085,439 | 25,112,221 | 92.71 | 24,263,428 | 96.62 | 19,770,449 |
| 0 dpi_F6 | 35,446,789 | 32,947,046 | 92.95 | 31,754,363 | 96.38 | 25,173,681 |
| 4dpi_F1 | 33,141,070 | 30,776,783 | 92.87 | 29,653,430 | 96.35 | 23,601,837 |
| 4dpi _F2 | 24,119,697 | 22,182,174 | 91.97 | 21,093,029 | 95.09 | 15,109,383 |
| 4dpi _F3 | 25,447,276 | 23,627,834 | 92.85 | 22,630,739 | 95.78 | 18,375,060 |
| 4dpi _F4 | 28,777,537 | 26,848,741 | 93.3 | 25,726,464 | 95.82 | 20,579,372 |
| 4dpi _F5 | 32,267,776 | 29,765,296 | 92.24 | 27,151,903 | 91.22 | 21,229,599 |
| 4dpi _F6 | 29,916,718 | 27,653,002 | 92.43 | 26,541,351 | 95.98 | 20,957,105 |
| 7dpi _F1 | 22,724,108 | 21,184,663 | 93.23 | 20,286,433 | 95.76 | 16,651,379 |
| 7dpi _F2 | 24,216,542 | 22,803,694 | 94.17 | 21,627,023 | 94.84 | 17,231,475 |
| 7dpi _F3 | 25,370,044 | 23,176,833 | 91.36 | 22,358,691 | 96.47 | 17,403,859 |
| 7dpi _F4 | 25,830,010 | 23,560,194 | 91.21 | 21,390,300 | 90.79 | 17,514,492 |
| 7dpi _F5 | 22,356,856 | 21,169,816 | 94.69 | 19,821,299 | 93.63 | 16,231,978 |
| 7dpi _F6 | 27,854,702 | 26,242,235 | 94.21 | 25,221,412 | 96.11 | 19,968,567 |
| 14dpi _F1 | 23,445,793 | 21,786,551 | 92.92 | 20,878,052 | 95.83 | 17,152,789 |
| 14dpi _F2 | 29,782,671 | 27,922,357 | 93.75 | 26,635,136 | 95.39 | 21,449,560 |
| 14dpi _F3 | 22,911,403 | 21,066,427 | 91.95 | 20,160,571 | 95.7 | 16,496,355 |
| 14dpi _F4 | 24,310,134 | 22,667,534 | 93.24 | 21,683,763 | 95.66 | 17,895,816 |
| 14dpi _F5 | 28,664,133 | 26,055,225 | 90.9 | 24,997,383 | 95.94 | 19,554,268 |
| 14dpi _F6 | 26,087,449 | 23,827,146 | 91.34 | 22,714,418 | 95.33 | 18,510,969 |
| 16dpi _F1 | 28,400,360 | 25,480,633 | 89.72 | 24,489,436 | 96.11 | 19,594,519 |
| 16dpi _F2 | 21,715,412 | 19,810,680 | 91.23 | 18,659,679 | 94.19 | 14,740,735 |
| 16dpi _F3 | 22,603,785 | 20,898,728 | 92.46 | 20,150,554 | 96.42 | 16,453,470 |
| 16dpi _F4 | 23,451,127 | 20,735,553 | 88.42 | 19,906,131 | 96 | 15,906,230 |
| 16dpi _F5 | 24,921,811 | 22,700,158 | 91.09 | 21,792,152 | 96 | 17,457,881 |
| 16dpi _F6 | 25,482,490 | 23,774,625 | 93.3 | 22,878,322 | 96.23 | 17,933,281 |


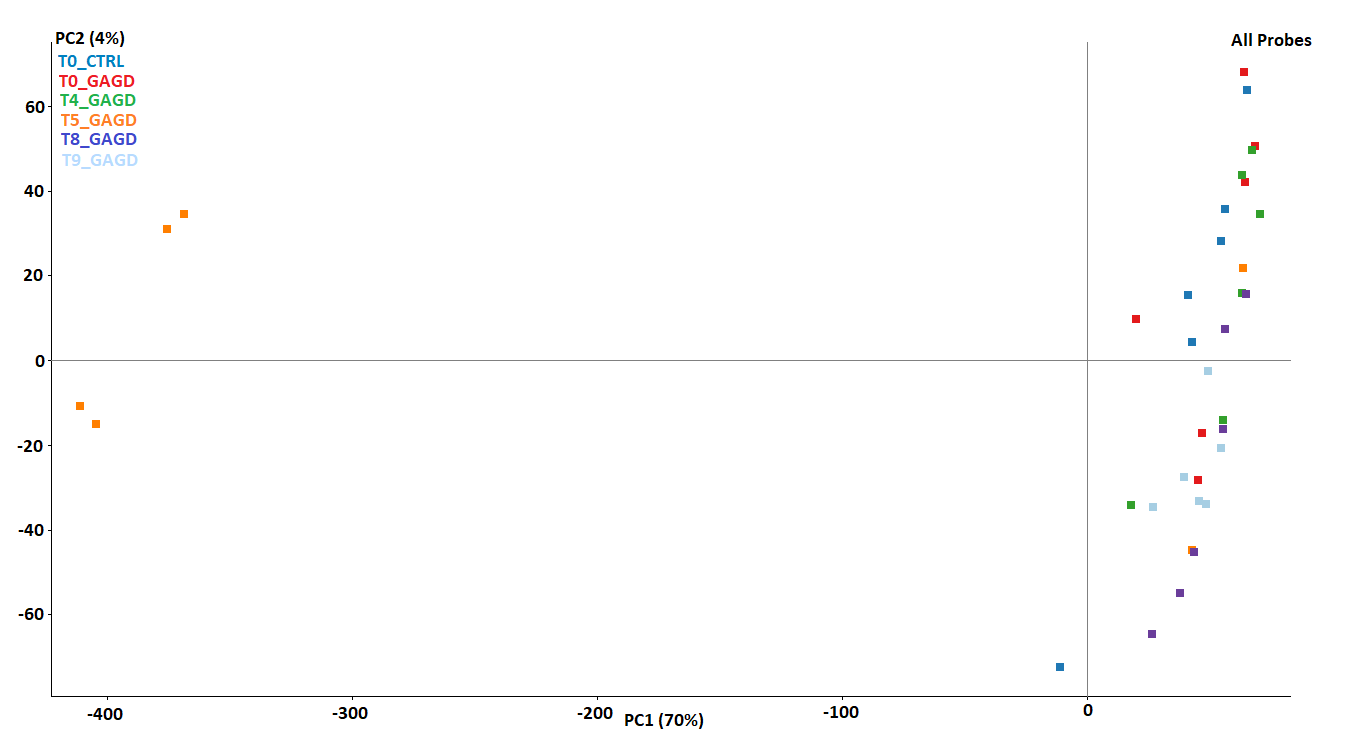


**Supplementary Figure S1.** Principal component analysis (PCA). The similarity between samples from thirty-six RNA-seq libraries (6 fish x 6 groups, including 2 groups from T0) was analysed. There were two groups of naïve control fish (T0_CTRL, T0_GAGD), one at 4 dpi (T4_GAGD), at 7 dpi (T5_GAGD), at 14 dpi (T8_GAGD) and at 16 dpi (T9_GAGD). The variation in the PC1 was 70%, and in PC2 was 4%. Four of the six AGD-affected samples at 7 dpi (orange squares) are clustered together. One 7 dpi sample is located in the quadrant with four 4 dpi samples (green squares) and the six 0 dpi control samples (blue squares), while the other 7 dpi sample is located with the 14 dpi (purple squares) and 16 dpi (light blue).

**Supplementary Table S2.** The top 20 down-regulated genes in AGD-affected gill at each time point.

GeneID: gene identification number, Log_2_FC: log of gene expression fold change to base 2, p-adj: p-value adjusted for false discovery rate, dpi: days post inoculation with *N. perurans*

|  | GeneID | Description | log_2_FC | p-adj |
| --- | --- | --- | --- | --- |
| 4 dpi | 106612333 | phytanoyl-CoA dioxygenase domain-containing protein 1-like | -5.79 | 0.000 |
|  | 106577151 | SH2 domain-containing protein 4B-like | -5.10 | 0.022 |
|  | 100136358 | nitric oxide synthase 2 | -4.97 | 0.000 |
|  | 106607306 | uncharacterized protein C17orf104 homolog | -4.41 | 0.000 |
|  | 106604090 | uncharacterized LOC106604090 | -4.11 | 0.000 |
|  | 106599381 | tandem C2 domains nuclear protein-like | -3.87 | 0.000 |
|  | 106580712 | neutrophil cytosol factor 2-like | -3.77 | 0.000 |
|  | 106561469 | E3 ubiquitin/ISG15 ligase TRIM25-like | -3.56 | 0.000 |
|  | 106600843 | interleukin-17F-like | -3.50 | 0.000 |
|  | 106612301 | uncharacterized LOC106612301 | -3.48 | 0.000 |
|  | 106575141 | GDP-Man:Man(3)GlcNAc(2)-PP-Dol alpha-1,2-mannosyltransferase-like | -3.46 | 0.032 |
|  | 100196393 | Macrophage inflammatory protein 2-alpha | -3.44 | 0.000 |
|  | 106609507 | poly [ADP-ribose] polymerase 12-like | -3.42 | 0.000 |
|  | 106572795 | eosinophil peroxidase-like | -3.20 | 0.000 |
|  | 106577820 | cytosolic phospholipase A2 gamma-like | -3.16 | 0.001 |
|  | 106566237 | 5-aminolevulinate synthase, nonspecific, mitochondrial-like | -3.04 | 0.000 |
|  | 106604810 | cytochrome b-245 heavy chain-like | -2.98 | 0.000 |
|  | 106581616 | aconitate decarboxylase 1 | -2.95 | 0.000 |
|  | 100136453 | cathelicidin | -2.95 | 0.000 |
|  | 106603239 | uncharacterized LOC106603239 | -2.85 | 0.000 |
| 7 dpi | 106582495 | interferon-induced guanylate-binding protein 1-like | -7.46 | 0.000 |
|  | 106581364 | uncharacterized LOC106581364 | -6.46 | 0.000 |
|  | 106612333 | phytanoyl-CoA dioxygenase domain-containing protein 1-like | -6.32 | 0.000 |
|  | 100136358 | nitric oxide synthase 2 | -5.76 | 0.000 |
|  | 106607306 | uncharacterized protein C17orf104 homolog | -5.39 | 0.000 |
|  | 106604090 | uncharacterized LOC106604090 | -5.10 | 0.000 |
|  | 106607322 | uncharacterized LOC106607322 | -4.78 | 0.000 |
|  | 106583660 | CD276 antigen-like | -4.45 | 0.000 |
|  | 106568225 | S100 calcium binding protein Z | -4.28 | 0.000 |
|  | 106610051 | NADPH oxidase organizer 1-like | -4.24 | 0.000 |
|  | 106561469 | E3 ubiquitin/ISG15 ligase TRIM25-like | -4.11 | 0.000 |
|  | 106572795 | eosinophil peroxidase-like | -4.03 | 0.000 |
|  | 106611403 | alpha-2C adrenergic receptor-like | -3.89 | 0.005 |
|  | 106580712 | neutrophil cytosol factor 2-like | -3.89 | 0.000 |
|  | 106562747 | uncharacterized LOC106562747 | -3.87 | 0.030 |
|  | 106577151 | SH2 domain-containing protein 4B-like | -3.83 | 0.040 |
|  | 106609025 | heterogeneous nuclear ribonucleoprotein C-like | -3.71 | 0.009 |
|  | 106583609 | uncharacterized LOC106583609 | -3.59 | 0.000 |
|  | 106581372 | uncharacterized LOC106581372 | -3.54 | 0.009 |
|  | 106603239 | uncharacterized LOC106603239 | -3.54 | 0.000 |
| 14 dpi | 106585533 | sphingomyelin phosphodiesterase 4-like | -5.52 | 0.026 |
|  | 106575141 | GDP-Man:Man(3)GlcNAc(2)-PP-Dol alpha-1,2-mannosyltransferase-like | -4.95 | 0.019 |
|  | 106610051 | NADPH oxidase organizer 1-like | -4.36 | 0.000 |
|  | 106612333 | phytanoyl-CoA dioxygenase domain-containing protein 1-like | -4.03 | 0.000 |
|  | 106607306 | uncharacterized protein C17orf104 homolog | -3.88 | 0.000 |
|  | 100136358 | nitric oxide synthase 2 | -3.55 | 0.000 |
|  | 106583107 | ephrin type-A receptor 8-like | -3.47 | 0.021 |
|  | 106607304 | uncharacterized LOC106607304 | -3.28 | 0.000 |
|  | 106607798 | solute carrier family 22 member 2-like | -3.20 | 0.031 |
|  | 106605128 | complement C3-like | -3.19 | 0.000 |
|  | 106604090 | uncharacterized LOC106604090 | -3.15 | 0.000 |
|  | 100196216 | microfibrillar-associated protein 4 | -3.08 | 0.010 |
|  | 106578717 | uncharacterized LOC106578717 | -3.00 | 0.000 |
|  | 106604696 | saxitoxin and tetrodotoxin-binding protein 2-like | -2.98 | 0.000 |
|  | 106611125 | apolipoprotein B-100-like | -2.97 | 0.021 |
|  | 106599381 | tandem C2 domains nuclear protein-like | -2.93 | 0.000 |
|  | 106570446 | polypeptide N-acetylgalactosaminyltransferase 12-like | -2.90 | 0.022 |
|  | 100196051 | Interleukin-20 receptor alpha chain | -2.85 | 0.000 |
|  | 106583788 | ATPase plasma membrane Ca2+ transporting 2 | -2.82 | 0.008 |
|  | 106581616 | aconitate decarboxylase 1 | -2.78 | 0.000 |
| 16 dpi | 106612333 | phytanoyl-CoA dioxygenase domain-containing protein 1-like | -5.82 | 0.000 |
|  | 100136358 | nitric oxide synthase 2 | -5.13 | 0.000 |
|  | 106599381 | tandem C2 domains nuclear protein-like | -5.07 | 0.000 |
|  | 106604090 | uncharacterized LOC106604090 | -5.02 | 0.000 |
|  | 106607306 | uncharacterized protein C17orf104 homolog | -4.82 | 0.000 |
|  | 106581616 | aconitate decarboxylase 1 | -4.34 | 0.000 |
|  | 106609507 | poly [ADP-ribose] polymerase 12-like | -4.23 | 0.000 |
|  | 106562747 | uncharacterized LOC106562747 | -3.89 | 0.048 |
|  | 106607304 | uncharacterized LOC106607304 | -3.87 | 0.000 |
|  | 106572795 | eosinophil peroxidase-like | -3.80 | 0.000 |
|  | 106577820 | cytosolic phospholipase A2 gamma-like | -3.72 | 0.001 |
|  | 106604696 | saxitoxin and tetrodotoxin-binding protein 2-like | -3.62 | 0.000 |
|  | 106603239 | uncharacterized LOC106603239 | -3.55 | 0.000 |
|  | 106561358 | fibroblast growth factor 23-like | -3.51 | 0.001 |
|  | 106602275 | hydroperoxide isomerase ALOXE3-like | -3.46 | 0.001 |
|  | 106600843 | interleukin-17F-like | -3.44 | 0.000 |
|  | 106585882 | C-C motif chemokine 4-like | -3.22 | 0.000 |
|  | 106606833 | uncharacterized LOC106606833 | -3.15 | 0.000 |
|  | 106580712 | neutrophil cytosol factor 2-like | -3.14 | 0.000 |
|  | 106580394 | L-serine dehydratase/L-threonine deaminase-like | -3.10 | 0.000 |

**Supplementary Table S3.** The top 20 up-regulated genes in AGD-affected gill at each time point.

GeneID: gene identification number, Log_2_FC: log of gene expression fold change to base 2, p-adj: p-value adjusted for false discovery rate, dpi: days post inoculation with *N. perurans*

|  | GeneID | Description | log_2_FC | p-adj |
| --- | --- | --- | --- | --- |
| 4 dpi | 106578657 | platelet-activating factor acetylhydrolase IB subunit gamma-like | 3.31 | 0.000 |
|  | 106561865 | calcium and integrin-binding family member 2-like | 2.90 | 0.000 |
|  | 106577212 | uncharacterized LOC106577212 | 2.76 | 0.000 |
|  | 106577163 | myelin and lymphocyte protein-like | 2.65 | 0.000 |
|  | 106566687 | transketolase-like | 2.63 | 0.000 |
|  | 106579988 | nuclear receptor subfamily 5 group A member 2-like | 2.62 | 0.000 |
|  | 106562982 | NK1 transcription factor-related protein 2-like | 2.62 | 0.000 |
|  | 106564214 | glycine-rich cell wall structural protein 1-like | 2.62 | 0.000 |
|  | 106563725 | uncharacterized LOC106563725 | 2.57 | 0.000 |
|  | 106608879 | protein inturned-like | 2.41 | 0.000 |
|  | 106613476 | deoxynucleotidyltransferase terminal-interacting protein 2-like | 2.35 | 0.000 |
|  | 106575639 | keratin, type I cytoskeletal 18-like | 2.32 | 0.017 |
|  | 106566079 | rho GTPase-activating protein 40-like | 2.29 | 0.000 |
|  | 106582792 | uncharacterized LOC106582792 | 2.26 | 0.000 |
|  | 106589400 | tartrate-resistant acid phosphatase type 5-like | 2.23 | 0.000 |
|  | 106574367 | trifunctional purine biosynthetic protein adenosine-3-like | 2.22 | 0.003 |
|  | 100195890 | Ly6/PLAUR domain-containing protein 2 | 2.20 | 0.000 |
|  | 106579989 | adhesion G-protein coupled receptor D2-like | 2.19 | 0.000 |
|  | 106611802 | saxitoxin and tetrodotoxin-binding protein 1-like | 2.18 | 0.026 |
|  | 106582757 | caM kinase-like vesicle-associated protein | 2.11 | 0.006 |
| 7dpi | 106572058 | mannan binding lectin serine peptidase 2 | 30.00 | 0.000 |
|  | 106577959 | butyrophilin subfamily 2 member A1-like | 29.63 | 0.000 |
|  | 106562200 | aquaporin 9 | 29.54 | 0.000 |
|  | 106578604 | uncharacterized LOC106578604 | 25.41 | 0.000 |
|  | 106574351 | ladderlectin-like | 25.04 | 0.000 |
|  | 106575496 | ladderlectin-like | 17.20 | 0.000 |
|  | 106605344 | uncharacterized LOC106605344 | 16.38 | 0.000 |
|  | 106577960 | butyrophilin subfamily 2 member A1-like | 16.09 | 0.000 |
|  | 106597213 | uncharacterized LOC106597213 | 16.06 | 0.000 |
|  | 106599177 | complement factor H-like | 16.00 | 0.000 |
|  | 106563718 | liver-expressed antimicrobial peptide 2-like | 15.85 | 0.000 |
|  | 106561249 | myo-inositol oxygenase | 15.77 | 0.000 |
|  | 106573520 | zona pellucida sperm-binding protein 4-like | 15.71 | 0.000 |
|  | 100380442 | Coagulation factor IX | 15.59 | 0.000 |
|  | 106606371 | uncharacterized LOC106606371 | 15.58 | 0.000 |
|  | 106598425 | angiopoietin-related protein 3-like | 15.55 | 0.000 |
|  | 100136583 | pentraxin | 15.54 | 0.000 |
|  | 106577777 | transmembrane 4 L six family member 5 | 15.53 | 0.000 |
|  | 106581172 | alpha-2-antiplasmin-like | 15.53 | 0.000 |
|  | 106608633 | serum amyloid P-component-like | 15.50 | 0.000 |
| 14 dpi | 106588483 | uncharacterized LOC106588483 | 4.33 | 0.000 |
|  | 106604507 | ribonuclease-like 3 | 4.14 | 0.000 |
|  | 106561635 | ladderlectin-like | 4.03 | 0.000 |
|  | 106588495 | uncharacterized LOC106588495 | 4.00 | 0.000 |
|  | 106564985 | uncharacterized LOC106564985 | 3.95 | 0.000 |
|  | 106564214 | glycine-rich cell wall structural protein 1-like | 3.71 | 0.000 |
|  | 106575633 | keratin, type I cytoskeletal 18-like | 3.70 | 0.007 |
|  | 106601707 | Ig heavy chain V region 914-like | 3.64 | 0.039 |
|  | 106564982 | uncharacterized LOC106564982 | 3.53 | 0.000 |
|  | 106564757 | keratin, type I cytoskeletal 19-like | 3.51 | 0.005 |
|  | 106583701 | protein-glutamine gamma-glutamyltransferase 2-like | 3.45 | 0.000 |
|  | 106575639 | keratin, type I cytoskeletal 18-like | 3.40 | 0.001 |
|  | 106562982 | NK1 transcription factor-related protein 2-like | 3.13 | 0.000 |
|  | 106586060 | uncharacterized LOC106586060 | 3.12 | 0.014 |
|  | 106563725 | uncharacterized LOC106563725 | 3.03 | 0.000 |
|  | 106561636 | ladderlectin-like | 3.01 | 0.008 |
|  | 106564983 | uncharacterized LOC106564983 | 3.01 | 0.000 |
|  | 106561865 | calcium and integrin-binding family member 2-like | 2.98 | 0.001 |
|  | 106589400 | tartrate-resistant acid phosphatase type 5-like | 2.97 | 0.000 |
|  | 106561862 | transient receptor potential cation channel subfamily M member 1-like | 2.96 | 0.010 |
| 16 dpi | 106583646 | neuronal acetylcholine receptor subunit alpha-2-like | 8.58 | 0.033 |
|  | 106588483 | uncharacterized LOC106588483 | 5.48 | 0.000 |
|  | 106588495 | uncharacterized LOC106588495 | 5.28 | 0.000 |
|  | 106583649 | uncharacterized LOC106583649 | 4.73 | 0.009 |
|  | 106586060 | uncharacterized LOC106586060 | 4.47 | 0.000 |
|  | 106575633 | keratin, type I cytoskeletal 18-like | 4.34 | 0.000 |
|  | 106575629 | intermediate filament protein ON3-like | 4.19 | 0.000 |
|  | 106590303 | trypsin-3-like | 4.14 | 0.000 |
|  | 106564757 | keratin, type I cytoskeletal 19-like | 3.97 | 0.000 |
|  | 106610823 | uncharacterized LOC106610823 | 3.93 | 0.000 |
|  | 106564214 | glycine-rich cell wall structural protein 1-like | 3.86 | 0.000 |
|  | 106563379 | endonuclease domain-containing 1 protein-like | 3.82 | 0.000 |
|  | 106575639 | keratin, type I cytoskeletal 18-like | 3.79 | 0.000 |
|  | 106561635 | ladderlectin-like | 3.54 | 0.000 |
|  | 106578030 | pentraxin fusion protein-like | 3.50 | 0.000 |
|  | 100195688 | Guanine nucleotide-binding protein GI/GS/GO subunit gamma-8 | 3.30 | 0.000 |
|  | 106561636 | ladderlectin-like | 3.30 | 0.003 |
|  | 100136455 | zonadhesin-like | 3.28 | 0.000 |
|  | 106605212 | potassium voltage-gated channel modifier subfamily G member 3 | 3.25 | 0.000 |
|  | 106576423 | muscarinic acetylcholine receptor M2-like | 4.94 | 0.000 |

**Supplementary Table S4.** Immune-related genes down-regulated genes at 7 dpi.

All genes listed were identified in R-DRE-1682256: Immune system denoted by^1^ of which subgroups included R-DRE-168249: Innate immune system^2^, R-DRE-1280215: Cytokine signalling in the immune system^3^, and R-DRE-449147: Signalling by interleukins^4^. GeneID: *Salmo salar* gene identification number

| Gene ID | Gene symbol | Name | Gene ID | Gene symbol | Name |
| --- | --- | --- | --- | --- | --- |
| 336425 | *aldoaa* | *aldolase a, fructose-bisphosphate, a*^1,2^ | 100537196 | *mapkap1* | *MAPK associated protein 1*^1^ |
| 114428 | *arf1* | *ADP-ribosylation factor* *1*^1^ | 373081 | *mvp* | *major vault protein*^1,2^ |
| 415204 | *arpc3* | *actin related protein 2/3 complex, subunit* 3^1,2^ | 569779 | *myo10* | *myosin X*^1,2^ |
| 767630 | *atp8a1* | *ATPase phospholipid transporting 8A1^1^*^,2^ | 557335 | *nlrx1* | *NLR family member X1*^1,2^ |
| 192322 | *calm2b* | *calmodulin 2b, (phosphorylase kinase, delta)* ^1,2^ | 60658 | *nos1* | *nitric oxide synthase 1 (neuronal)* ^1,2^ |
| 334527 | *cap1* | *CAP, adenylate cyclase-associated protein* 1^1,2^ | 404036 | *nos2a* | *nitric oxide synthase 2a, inducible*^1,2^ |
| 394037 | *cct8* | *chaperonin containing TCP1, subunit 8 (theta)*^1,2^ | 796461 | *nrg1* | *neuregulin 1*^1, 3, 4^ |
| 567192 | *cd59* | *CD59 molecule (CD59 blood group*)^1,2^ | 554967 | *psmd1* | *proteasome 26S subunit, non-ATPase 1*^1,2^ |
| 767754 | *cenpe* | *centromere protein E^1^* | 373104 | *rab14* | *RAB14, member RAS oncogene family*^1,2^ |
| 336381 | *cotl1* | *coactosin-like F-actin binding protein 1^1^*^,2^ | 323197 | *racgap1* | *Rac GTPase activating protein 1*^1^ |
| 100134935 | *csf3r* | *colony stimulating factor 3 receptor (granulocyte)*^1, 3, 4^ | 554089 | *s100z* | *S100 calcium binding protein Z*^1,2^ |
| 30265 | *ctnnb1* | *catenin (cadherin-associated protein), beta 1*^1^ | 402992 | *scamp1* | *secretory carrier membrane protein 1*^1,2^ |
| 100002946 | *cxcl8a* | *chemokine (C-X-C motif) ligand 8a*^1^ | 793290 | *spred2b* | *sprouty related EVH1 domain containing 2b*^1, 3, 4^ |
| 336613 | *cyfip1* | *cytoplasmic FMR1 interacting protein 1*^1,2^ | 368519 | *stat4* | *signal transducer and activator of transcription 4*^1,^ ^3, 4^ |
| 324089 | *det1* | *DET1 partner of COP1*^1^ | 492489 | *sugt1* | *SGT1 homolog, MIS12 kinetochore complex assembly cochaperone*^1,2^ |
| 114446 | *dlg1* | *discs, large homolog 1 (Drosophila)* ^1, 3, 4^ | 405769 | *syk* | *spleen tyrosine kinase*^1, 2, 3, 4^ |
| 100005297 | *epgn* | *epithelial mitogen homolog (mouse)* ^1, 3, 4^ | 100333043 | *tap1* | *transporter 1, ATP-binding cassette, sub-family B (MDR/TAP)* ^1^ |
| 562999 | *hectd2* | *HECT domain containing*^1^ | 692289 | *tbk1* | *TANK-binding kinase 1*^1, 2, 3, 4^ |
| 30573 | *hsp90ab1* | *heat shock protein 90, alpha (cytosolic), class B member 1*^1,2^ | 100333821 | *tnfrsf11a* | *TNF receptor superfamily, member 11a, NFKB activator*^1,3^ |
| 563560 | *ikbkb* | *inhibitor of nuclear factor kappa B kinase subunit beta*^1, 2, 3^ | 564279 | *tnip2* | *TNFAIP3 interacting protein 2*^1, 2, 3^ |
| 560193 | *Il-34* | *interleukin 34*^1, 3, 4^ | 100331669 | *tpp2* | *tripeptidyl peptidase 2*^1^ |
| 403013 | *irf9* | *interferon regulatory factor 9*^1, 3, 4^ | 406335 | *uba1* | *ubiquitin-like modifier activating enzyme 1*^1^ |
| 561370 | *jak3* | *Janus kinase 3 (a protein tyrosine kinase, leukocyte)*^1, 3, 4^ | 393934 | *ube2d2* | *ubiquitin-conjugating enzyme E2D 2 (UBC4/5 homolog, yeast)* ^1,2^ |
| 555969 | *lpcat1* | *lysophosphatidylcholine acyltransferase 1*^1,2^ | 406807 | *ube2na* | *ubiquitin-conjugating enzyme E2Na*^1 ,2, 3, 4^ |
| 399480 | *mapk3* | *mitogen-activated protein kinase 3* ^1, 2, 3, 4^ | 321056 | *zgc:63569* | *zgc:63569*^1,2^ |
|  |  |  | 393844 | *znrf1* | *zinc and ring finger 1*^1^ |

**Supplementary Table S5**. Relative Gene Expression of interferon-related genes in AGD-affected gill. Gene symbols include *ifn; ifn, irf: ifn regulatory factor, ifng: ifn gamma,* dpi: days post inoculation, GeneID: *Salmo salar* gene identification number, chr: chromosome location, p-adj: P-values adjusted for false discovery rate, log_2_FC: log of gene expression fold change to base 2. Up-regulated genes are highlighted with bold text.

| Time | GeneID | Gene symbol | Chr | p-adj | log_2_FC |
| --- | --- | --- | --- | --- | --- |
| 4 dpi | 106564725 | *ifngr1b* | ssa12 | 0.00 | -0.8 |
|  | 106581880 | *ifngr2a* | ssa21 | 0.01 | -0.8 |
|  | 100136548 | *irf1* | ssa13 | 0.04 | -0.4 |
|  | 100337625 | *irf3* | ssa19 | 0.00 | -0.5 |
|  | 106565674 | *irf6-like* | ssa12 | 0.03 | -0.5 |
|  | 100329194 | *irf 7B* | ssa10 | 0.00 | -0.8 |
| 7 dpi | 106581896 | *Ifnr(a/b)1a-like* | ssa21 | 0.03 | -1.3 |
|  | 106587463 | *ifngr1a* | ssa02 | 0.01 | -1.5 |
|  | 106564725 | *Ifngr1b* | ssa12 | 0.01 | -1.9 |
|  | 106581880 | *irf2a* | ssa21 | 0.01 | -1.2 |
|  | 100136548 | *irf1* | ssa13 | 0.01 | -1.6 |
|  | 100846970 | *irf1 isoform 2* | ssa04 | 0.03 | -1.0 |
|  | 100136507 | *irf2* | ssa09 | 0.04 | -0.6 |
|  | **106612476** | ***irf2-like*** | **ssa01** | **0.00** | **2.5** |
|  | 106566745 | *irf4-like* | ssa13 | 0.02 | -1.6 |
|  | 100194823 | *irf5* | ssa17 | 0.00 | -0.8 |
|  | 100329194 | *irf7B* | ssa10 | 0.00 | -2.1 |
|  | 106562284 | *irf8-like* | ssa11 | 0.03 | -0.8 |
|  | 100380435 | *irf9* | ssa19 | 0.00 | -0.6 |
| 14 dpi | **106612476** | ***irf 2-like*** | **ssa01** | **0.04** | **0.8** |
|  | 100337625 | *irf 3* | ssa19 | 0.01 | -0.4 |
|  | **100194953** | *irf* ***4*** | **ssa23** | **0.03** | **0.4** |
|  | **106599909** | ***irf 4-like*** | **ssa03** | **0.01** | **0.6** |
|  | **106571913** | ***irf 4-like*** | **ssa15** | **0.00** | **0.6** |
|  | 100329194 | *irf 7B* | ssa10 | 0.00 | -0.8 |
| 16 dpi | 106600865 | *ifn a3* | ssa03 | 0.00 | -1.3 |
|  | 106564725 | *ifngr 1b* | ssa12 | 0.02 | -0.4 |
|  | 100136548 | *irf 1* | ssa13 | 0.00 | -0.7 |
|  | 100337625 | *irf 3* | ssa19 | 0.00 | -0.8 |
|  | 106566745 | *irf 4-like* | ssa13 | 0.05 | -0.6 |
|  | 106609132 | *irf 5-like* | ssa07 | 0.02 | -0.5 |
|  | 100329194 | *irf 7B* | ssa10 | 0.00 | -1.4 |
|  | 100380435 | *irf 9* | ssa19 | 0.00 | -1.6 |

**Supplementary Table S6.** Relative Gene Expression of TNF and TGF-β in AGD-affected gill.

Gene symbols: *tnf-α: tumor necrosis factor-alpha, tgf-β:* *transforming growth factor-beta*

dpi: days post inoculation, GeneID: *Salmo salar* gene identification number, chr: chromosome location, p-adj: P-values adjusted for false discovery rate, log_2_FC: log of gene expression fold change to base 2. Up-regulated genes are highlighted with bold text.

| Time | GeneID | Gene symbol | Chr | p-adj | Log_2_FC |
| --- | --- | --- | --- | --- | --- |
| 4 dpi | 100136457 | *tnf-α1 precursor* | ssa05 | 0.0002 | -1.4 |
|  | 100136458 | *tnf-α2 precursor* | ssa02 | 0.0291 | -1.0 |
| 7 dpi | 100136509 | *tnf-α* | ssa14 | 0.0296 | -3.0 |
|  | 100136457 | *tnf-α1 precursor* | ssa05 | 0.0016 | -1.6 |
|  | 100136458 | *tnf-α2 precursor* | ssa02 | 0.0034 | -1.7 |
|  | 100195362 | ***tgf-β induced*** | ssa09 | 0.0001 | **0.9** |
|  | 106584161 | ***tgf-β2-like*** | ssa02 | 0.0000 | **3.2** |
|  | 106590795 | ***tgf-β3-like*** | ssa01 | 0.0049 | **1.0** |
| 14 dpi | 100136457 | *tnf-α1 precursor* | ssa05 | 0.0038 | -1.2 |
|  | 106569003 | ***tgf-β1-like*** | ssa14 | 0.0325 | **1.3** |
| 16 di | 106569003 | ***tgf-β1-like*** | ssa14 | 0.0001 | **2.1** |
|  | 106584161 | ***tgf-β2-like*** | ssa02 | 0.0144 | **0.7** |

**Supplementary Table S7**. Relative Gene Expression of *mucin (muc*) gene family members in gill tissue following *N. perurans* exposure. dpi: days post inoculation, GeneID: gene identification number, chr: chromosome location, p-adj: P-values adjusted for false discovery rate, Log_2_FC: log of gene expression fold change to base 2. Up-regulated genes are highlighted with bold text

| Time | GeneID | Gene symbol | Chr | p-adj | Log_2_FC |
| --- | --- | --- | --- | --- | --- |
| 4 dpi | 106608496 | *muc-2-like* | ssa07 | 0.0000 | -0.8 |
|  | 106612949 | ***muc-5AC-like*** | ssa09 | 0.0072 | **1.2** |
|  | 106560392 | *muc-7-like* | ssa10 | 0.0000 | -1.7 |
| 7 dpi | 106608493 | *muc-2-like* | ssa07 | 0.0235 | -2.5 |
|  | 106603020 | *muc-2-like* | ssa04 | 0.0235 | -1.8 |
|  | 106613952 | *muc-2-like* | ssa10 | 0.0041 | -0.8 |
|  | 106584523 | ***muc-2-like*** | ssa23 | 0.0000 | **3.0** |
|  | 106577588 | *muc-5AC-like* | ssa18 | 0.0444 | -0.6 |
|  | 106560392 | *muc-7-like* | ssa10 | 0.0073 | -2.6 |
|  | 106579100 | ***muc-13-like*** | ssa19 | 0.0000 | **7.8** |
|  | 106607023 | *muc-17-like* | ssa06 | 0.0361 | -0.7 |
|  | 106585310 | *muc-17-like* | ssa24 | 0.0268 | -0.6 |
| 14 dpi | 106560392 | *muc-7-like* | ssa10 | 0.0000 | -1.3 |
|  | 106565164 | ***muc-12-like*** | ssa12 | 0.0007 | **0.9** |
| 16 dpi | 106560392 | *muc-7-like* | ssa10 | 0.0000 | -1.5 |
|  | 106561817 | ***intestinal muc-like*** | ssa10 | 0.0000 | **0.8** |

**Supplementary Table S8.** . Primer sequences for quantitative PCR validation of Atlantic salmon immune differentially expressed genes in response to AGD. Genes included *acod1: aconitate decarboxylase 1* (also known as *irg1*), *cath1: cathelicidin 1*, *clra*: *c type lectin receptor a*, *cxc4: c-c motif chemokine 4-like*, *lect2: leukocyte cell-derived chemotaxin 2-like*, *il-8: interleukin-8-like*, *interleukin-17*: *il-17f-like*, *nos: nitric oxide synthase 2, inducible*, *ptx: pentraxin-related protein-like*, *sap: serum amyloid P-component-like*. All primers were designed to have Tm of 62^o^C

| Gene | Gene ID | Accession No. | Forward Primer | Reverse primer | Amplicon size |
| --- | --- | --- | --- | --- | --- |
| 106581616 | *acod* | XM_014163747.1 | ctccagtacagccagttcttca | cagggtgccacgtatcatca | 142 |
| 106585882 | *cxc4* | XM_014172601.1 | gcagcagcattctcagaggt | gtctcacccctgtcttggtg | 157 |
| 106600843 | *il-17a/f1* | XM_014192555.1 | gactaccaacaccacctatg | ccaggatctggtggtaaatg | 140 |
| 106577833 | *il-8* | XM_014156195.1 | tgctctaaggaagaagtcattgtca | gttgattctgacgctgttgattt | 165 |
| 106611589 | *lect2* | XM_014211962.1 | ggtgctcatagctgtgttgtc | tacacacaatgtccaggccc | 161 |
| 106581433 | *ptx3* | XM_014163494.1 | actggagcccacagcta | aagcatgttctccctcatctg | 112 |
| 106604759 | *sap* | XM_014199767.1 | gtgtaggccgtcactacctc | tggaaaacgccctcttgaca | 150 |
| 100136453 | *cath1* | NM_001123586.1 | aaatgggaaactaatgatgtgcagc | ggagcattttctggcttggc | 163 |
| 100136446 | *clra* | NM_001123581.1 | actgctgaggaacagacgtt | cgtcaatgtggttgttgggc | 169 |
| 100136525 | ef1aα | NM_001123629.1 | gctgctgagatgggtaa | caaacttccacagggaaatg | 133 |
| 100136358 | *nos2* | XM_014214975.1 | tctcttcctgggctctcacc | gaaagtgctgtgatgcggtc | 169 |
